# Supplementary material for: Highly efficient synthesis of non-planar macrocycles possessing intriguing self-assembling behaviors and ethene/ethyne capture properties
Source: Nat Commun. 2020 Nov 16;11:5806. doi: 10.1038/s41467-020-19677-x (PMC7669899; doi:10.1038/s41467-020-19677-x)
Supplement: Supplementary file 1 — Supplementary Information [file 41467_2020_19677_MOESM1_ESM.pdf]

Supplementary Information

Highly efficient synthesis of non-planar macrocycles possessing intriguing self-assembling behaviors and ethene/ethyne capture properties

Mao et al

Contents

Section A. Supplementary Materials and Methods 2
Section B. Synthesis and characterization of **DPA[n]s** 2
Section C. Chirality of **DPA[3]** 5
Section D. Molecular modeling of **DPA[n]s** 7
Section E. Aggregation behavior of **DPA[n]s** 9
Section F. Host-guest interaction 21
Section G. X-ray crystal data 27
Section H. Characterization data and spectra for **DPA[n]s** 35
Section I. Reference 45

Section A. Supplementary Materials and Methods

All solvents were dried according to the standard procedures and all of them were degassed under N_2 for 30 minutes before use. All air-sensitive reactions were carried out under inert N_2 atmosphere. ^{1}H and ^{13}C NMR spectra were recorded at 400 MHz with a Mercury plus 400 spectrometer at 298 K and tetramethylsilane (TMS) as an internal reference. The ^{1}H and ^{13}C NMR chemical shifts are reported relative to the residual solvent signals. Coupling constants (J) are denoted in Hz and chemical shifts (δ) in ppm. Multiplicities are denoted as follows: s = singlet, d = doublet. 2D NMR spectra (NOESY and DOSY) and variable-temperature ^{1}H NMR spectra were recorded on Bruker 500 MHz Spectrometer at 298 K. Mass spectra were recorded with Thermo Scientific LTQ XL spectrometer with methanol or acetonitrile as solvents. For the single crystals, the data sets were treated with the SQUEEZE program to remove highly disordered solvent molecules. The crystallographic formulae include the number of solvent molecules was suggested by the SQUEEZE program. Scanning electron microscopy (SEM) was performed on a Hitachi S-4800 microscope. The AFM samples were prepared by drop casting method using mica sheet as substrate. All the AFM images were obtained on a Dimension FastScan (Bruker), using ScanAsyst mode under ambient condition.

Section B. Synthesis and characterization of DPA[n]s

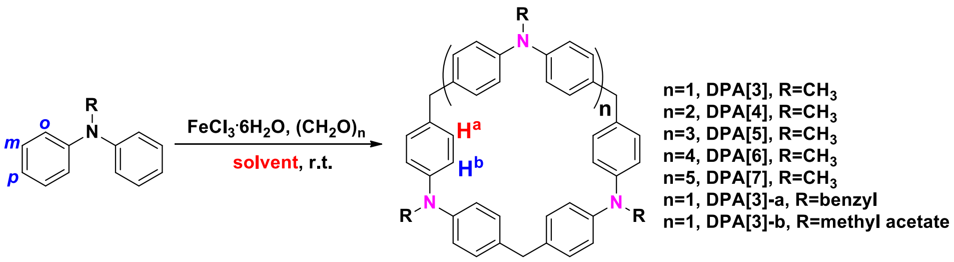

Supplementary Figure 1. Synthetic route to **DPA[n]s** (n = 3-7).

DPA[n]: To the solution of N-methyldiphenylamine (1.00 g, 5.46 mmol) in dichloromethane (500 mL) was added paraformaldehyde (492 mg, 16.4 mmol). The suspension was stirred at 25 °C for 30 min to crush the large paraformaldehyde particles. And then, $FeCl_3 \cdot 6H_2O$ (1.48 g, 5.46 mmol) was added to the solution. After continuing stirring at room temperature for 6 h, the reaction was quenched by addition of water. The organic phase was separated and washed with saturated aqueous $NaHCO_3$, H_2O, and brine. The crude product was purified by column chromatograph (CH_2Cl_2/petroleum ether) to yield **DPA[3]** (white solid, CH_2Cl_2/petroleum ether = 1:1, R_f = 0.3, 213 mg, 20%), **DPA[4]** (white solid, CH_2Cl_2/petroleum ether = 2:1, R_f = 0.3, 107 mg, 10%), **DPA[5]** (white solid, CH_2Cl_2/petroleum ether = 3:1, R_f = 0.3, 10.7 mg, 1%), **DPA[6]** (white solid, CH_2Cl_2/petroleum ether = 4:1, R_f = 0.3, 5.3 mg, 0.5%), **DPA[7]** (white solid, CH_2Cl_2, R_f = 0.3, 3.0 mg, 0.3%).

DPA[3]: ^{1}H NMR (400 MHz, $CDCl_3$): δ 7.04 (d, J = 8.0 Hz, 4H), 6.96 (d, J = 8.0 Hz, 4H), 3.96 (s, 2 H), 3.31 (s, 3H). ^{13}C NMR (100 MHz, $CDCl_3$): δ 147.2, 134.7, 129.8, 120.8, 40.6, 39.9. HR-ESI-TOF MS: Calcd. for $[M+H]^+$: 586.3222. Found: 586.3237.

DPA[4]: ^{1}H NMR (400 MHz, $CDCl_3$): δ 7.07 (d, J = 8.0 Hz, 4H), 6.96 (d, J = 8.0 Hz, 4H), 3.84 (s, 2 H), 3.27 (s, 3H). ^{13}C NMR (100 MHz, $CDCl_3$): δ 147.2, 134.7, 129.8, 120.8, 40.6, 39.9. HR-ESI-TOF MS: Calcd. for $[M+H]^+$: 781.4170. Found: 781.4143.

DPA[5]: ^{1}H NMR (400 MHz, $CDCl_3$): δ 7.07 (d, J = 8.0 Hz, 4H), 6.92 (d, J = 8.0 Hz, 4H), 3.84 (s, 2H), 3.26 (s, 3H). ^{13}C NMR (100 MHz, $CDCl_3$): δ 147.2, 134.3, 129.5, 120.4, 40.5, 39.4. HR-ESI-TOF MS: Calcd. for $[M+H]^+$: 976.5318. Found: 976.5320.

DPA[6]: ^{1}H NMR (400 MHz, $CDCl_3$): δ 7.07 (d, J = 8.0 Hz, 4H), 6.92 (d, J = 8.0 Hz, 4H), 3.85 (s, 2H), 3.26 (s, 3H). ^{13}C NMR (100 MHz, $CDCl_3$): δ 147.2, 134.3, 129.6, 120.4, 40.5, 40.4. HR-ESI-TOF MS: Calcd. for $[M+H]^+$: 1171.6366. Found: 1171.6363.

DPA[7]: ^{1}H NMR (400 MHz, $CDCl_3$): δ 7.07 (d, J = 8.0 Hz, 4H), 6.93 (d, J = 8.0 Hz, 4H), 3.84 (s, 2 H), 3.25 (s, 3H). ^{13}C NMR (100 MHz, $CDCl_3$): δ 147.2, 134.3, 129.5, 120.4, 40.5, 40.4. HR-ESI-TOF MS: Calcd. for $[M+H]^+$: 1367.7448. Found: 1367.7428.

DPA[3]-a: To the solution of N-benzyl-N-phenylaniline (1.00 g, 3.86 mmol) in dichloromethane (500 mL) was added paraformaldehyde (347 mg, 11.6 mmol). The suspension was stirred at 25 ℃ for 30 min to crush the large paraformaldehyde particles. And then, $FeCl_3•6H_2O$ (1.04 g, 3.86 mmol) was added to the solution. After continuing stirring at room temperature for 6 h, the reaction was quenched by addition of water. The organic phase was separated and washed with saturated aqueous $NaHCO_3$, H_2O, and brine. The crude product was purified by column chromatograph (CH_2Cl_2/petroleum ether) to yield **DPA[3]-a** (white solid, CH_2Cl_2/petroleum ether = 3:1, R_f = 0.3, 889 mg, 85%). ^{1}H NMR (400 MHz, $CDCl_3$): δ 7.36 (d, J = 7.4 Hz, 2H), 7.29 (t, J = 7.5 Hz, 2H), 7.21 (t, J = 7.3 Hz, 1H), 6.97 (d, J = 8.6 Hz, 4H), 6.92 (d, J = 8.6 Hz, 4H), 4.93 (s, 2H), 3.88 (s, 2H). ^{13}C NMR (100 MHz, $CDCl_3$): δ 149.1, 141.8, 136.7, 132.4, 131.0, 129.3, 123.6, 59.1, 42.2. HR-ESI-TOF MS: Calcd. for $[M+H]^+$: 814.4161. Found: 814.4133.

DPA[3]-b: To the solution of methyl diphenylglycinate (1.00 g, 4.14 mmol) in dichloromethane (500 mL) was added paraformaldehyde (373 mg, 12.4 mmol). The suspension was stirred at 25 ℃ for 30 min to crush the large paraformaldehyde particles. And then, $FeCl_3•6H_2O$ (1.12 g, 4.14 mmol) was added to the solution. After continuing stirring at room temperature for 6 h, the reaction was quenched by addition of water. The organic phase was separated and washed with saturated aqueous

$NaHCO_3$, H_2O, and brine. The crude product was purified by column chromatograph (CH_2Cl_2/petroleum ether) to yield **DPA[3]-b** (white solid, CH_2Cl_2/petroleum ether = 5:1, R_f = 0.3, 472 mg, 45%). ^{1}H NMR (400 MHz, $CDCl_3$): δ 7.00 (d, J = 8.0 Hz, 4H), 6.86 (t, J = 8.0 Hz, 4H), 4.40 (s, 2H), 3.93 (s, 2H), 3.72 (s, 3H). ^{13}C NMR (100 MHz, $CDCl_3$): δ 171.8, 146.4, 135.1, 130.2, 121.3, 52.3, 40.0. HR-ESI-TOF MS: Calcd. for $[M+H]^+$: 760.3387. Found: 760.3400.

Supplementary Table 1. Optimization of **DPA[n]s**.

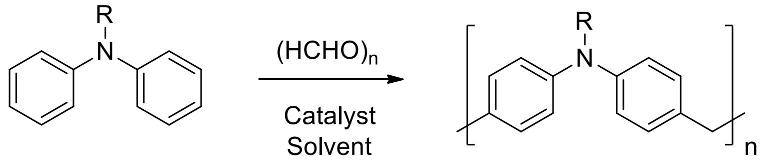

entry	R group	solvent	catalyst	time/ h	yield (%)
1	CH_3	dichloromethane	$FeCl_3$•$6H_2O$	6	**DPA[3]** (20%)<br>**DPA[4]** (10%)<br>**DPA[5]** (1%)<br>**DPA[6]** (0.5%)<br>**DPA[7]** (0.3%)
2	CH_3	dichloromethane	$FeCl_3$•$6H_2O$	12	**DPA[3]** (55%)<br>**DPA[4]** (5%)
3	CH_3	1,2-dichloroethane	$FeCl_3$•$6H_2O$	6	**DPA[3]** (50%)<br>**DPA[4]** (< 1%)
4	CH_3	1,2-dichloroethane	$FeCl_3$•$6H_2O$	12	**DPA[3]** (75%)<br>**DPA[4]** (< 1%)
5	CH_3	dichloromethane	$FeCl_3$	6	**DPA[3]** (10%)<br>**DPA[4]** (< 1%)
6	CH_3	dichloromethane	$AlCl_3$	6	**DPA[3]** (5%)<br>**DPA[4]** (< 1%)
7	CH_3	dichloromethane	BF_3•OEt_2	6	no macrocycle
8	CH_3	dichloromethane	TsOH	6	no macrocycle
9	CH_3	dichloromethane	H_2SO_4	6	no macrocycle
10	CH_3	dichloromethane	H_3PO_4	6	no macrocycle
11	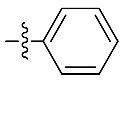	dichloromethane	$FeCl_3$•$6H_2O$	6	no macrocycle
12	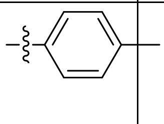	dichloromethane	$FeCl_3$•$6H_2O$	6	no macrocycle
13	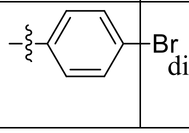	dichloromethane	$FeCl_3$•$6H_2O$	6	no macrocycle

14	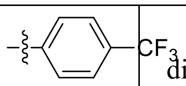	dichloromethane	$FeCl_3 \cdot 6H_2O$	6	no macrocycle

Section C. Chirality of DPA[3]

1. Rotation barriers of DPA[3]

To find out the energy barrier between optical isomers, structure of the transition state (***TS*-DPA[3]**) was obtained by the synchronous transit-guided Quasi-Newton (STQN) method[1,2] with the QST3 option. All calculations were performed by the Gaussian 16 software package[3].

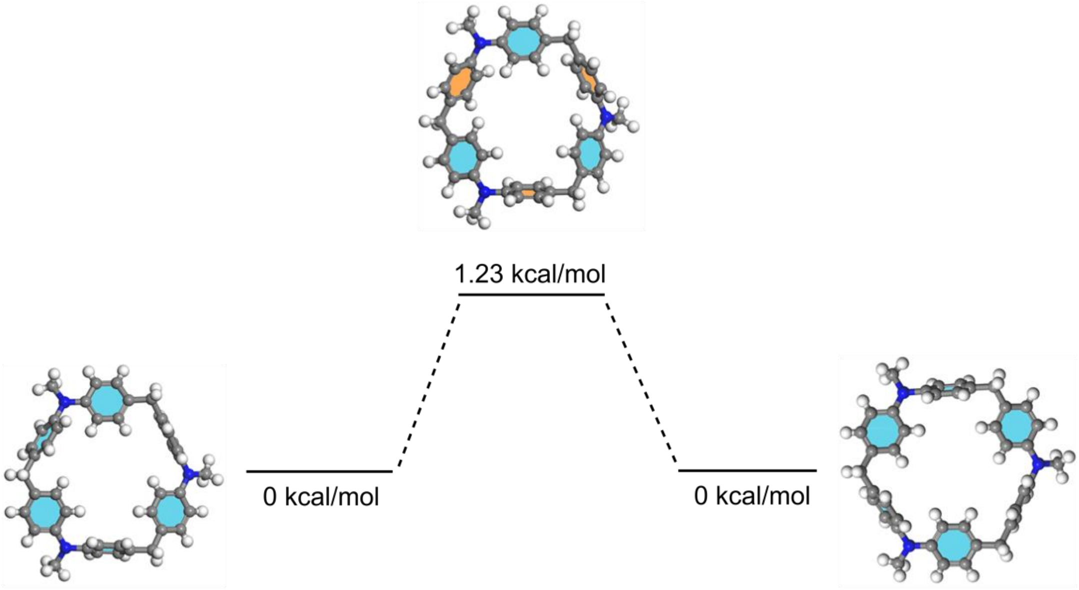

Supplementary Figure 2. Energy diagram of the inversion process of **DPA[3].**

2. Variable-temperature ^{1}H NMR experiment

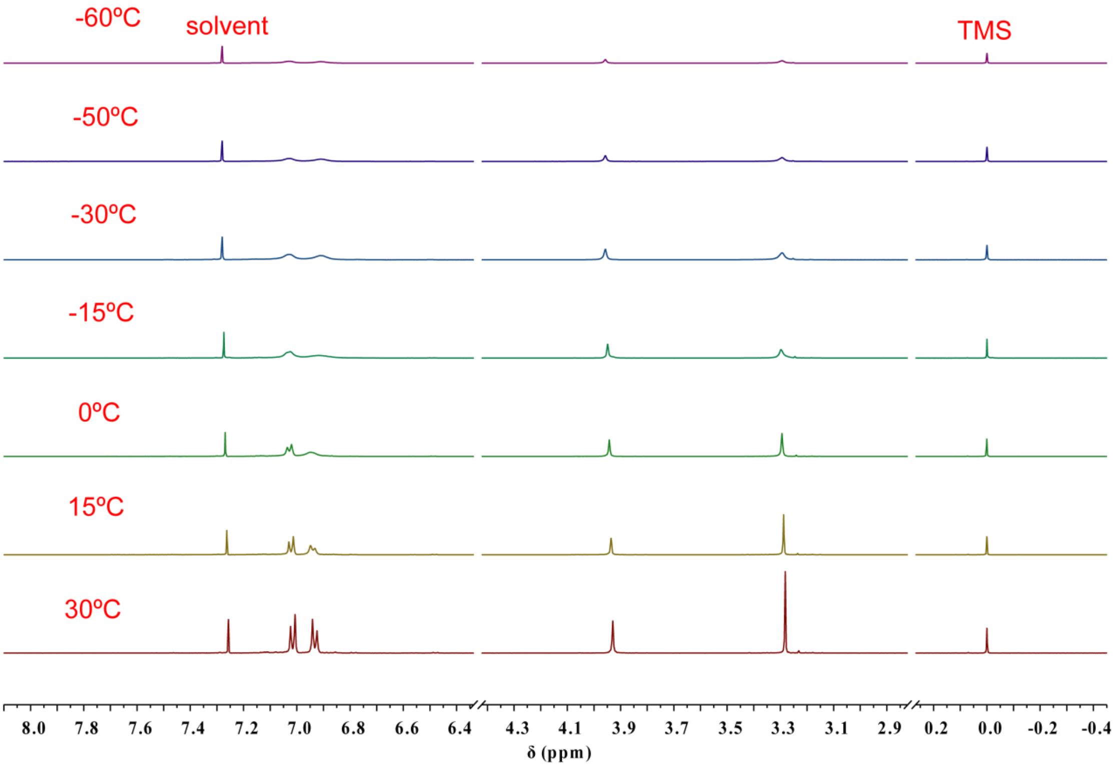

Supplementary Figure 3. Partial stacked variable-temperature ^{1}H NMR (500 MHz) spectra of **DPA[3]** (10 mM) in $CDCl_3$.

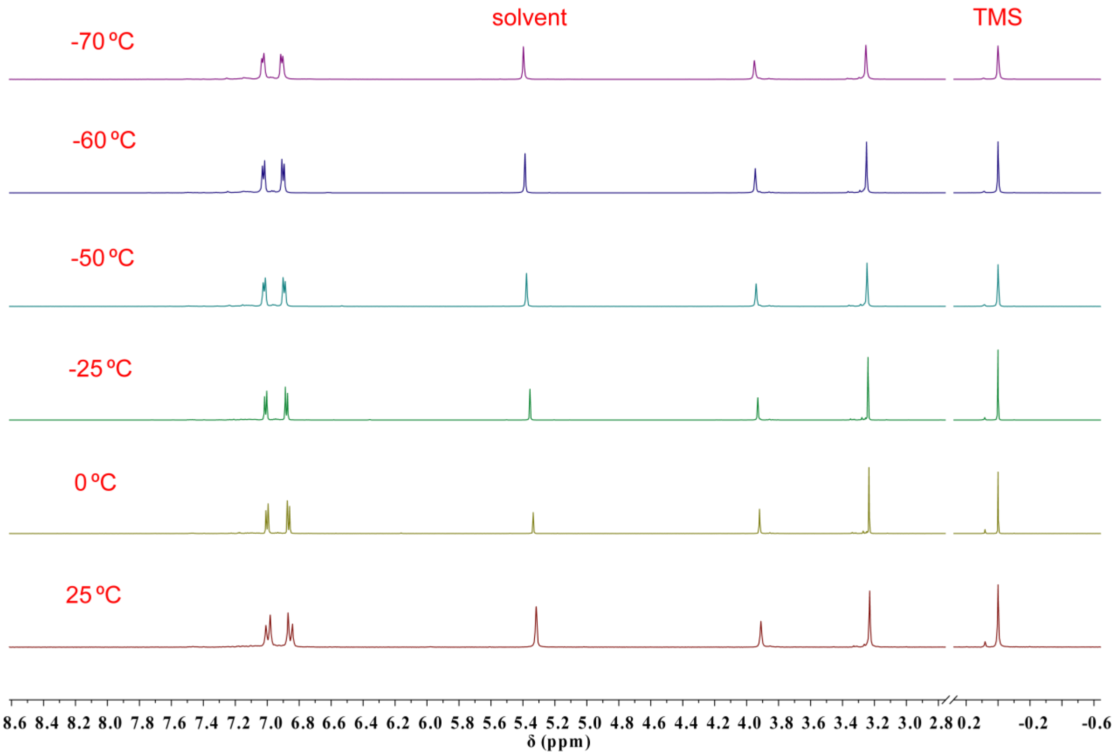

Supplementary Figure 4. Partial stacked variable-temperature ^{1}H NMR (500 MHz) spectra of **DPA[3]** (10 mM) in CD_2Cl_2.

Section D. Molecular modeling of DPA[n]s

1. DFT calculation of DPA[n]s

Geometries of molecules **DPA[n]s** were optimized at B3LYP/cc-pVDZ level with Grimme's dispersion correction[4] followed by frequency calculations to confirm the stationary points. All calculations were performed by the Gaussian 16 software package[3].

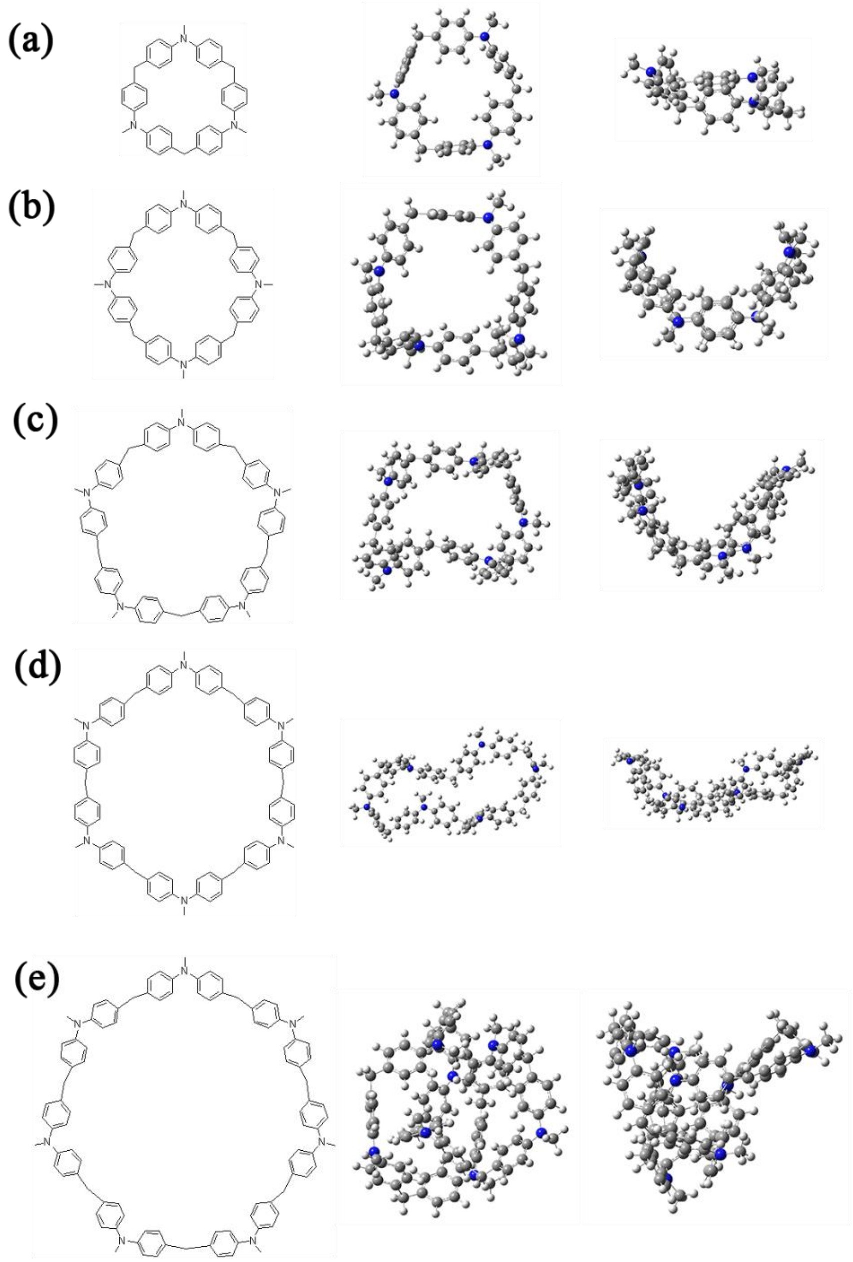

Supplementary Figure 5. Chemical structures of **DPA[3]** (a), **DPA[4]** (b), **DPA[5]** (c), **DPA[6]** (d), and **DPA[7]** (e) and their view of geometry optimized by RB3LYP/cc-pVDZ.

2. HOMO and LUMO orbitals of DPA[n]s

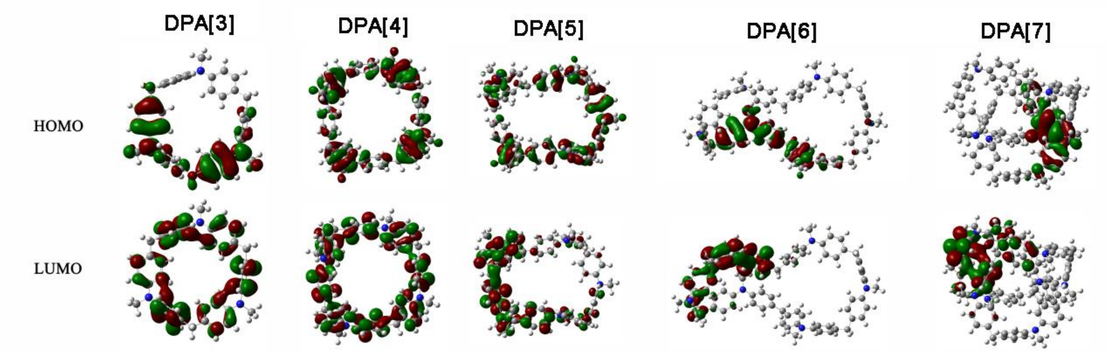

Supplementary Figure 6. Calculated molecular HOMO and LUMO orbitals of **DPA[n]s**.

3. Binding energies of DPA[n]s dimers

The binding energies were calculated at B97D3/cc-pVDZ level for a few theoretically predicted packing arrangements. Crystal packing arrangements were listed in Supplementary Tables 2 and 3 for **DPA[3]** and **DPA[4]**, respectively. To compare with the experimental arrangements, theoretically predicted structures have lower binding energies, indicating that experimental packing is more stable than the predicted packing arrangements.

Supplementary Table 2. Binding energies, E_b (kcal/mol) of different packing arrangements for **DPA[3]**.

packing	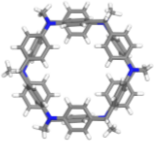 (from single crystal)	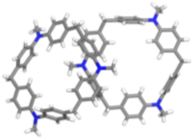	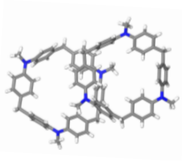	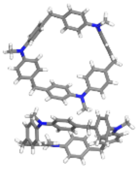	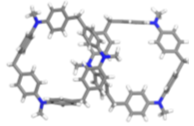
E_b	38.9	9.2	8.5	7.7	6.3

Supplementary Table 3. Binding energies, E_b (kcal/mol) of different packing arrangements for **DPA[4]**.

packing	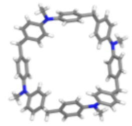 (from single crystal)	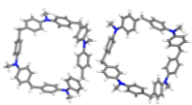	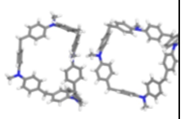	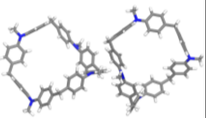	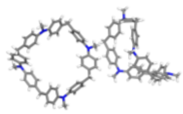
E_b	57.4	6.7	4.6	2.1	1.0

Section E. Aggregation behavior of DPA[n]s

1. NMR analysis of self-assembly

We analyzed the self-assembly of **DPA[n]** by using a curve-fitting method based on the isodesmic model under the equal K_E (EK model) assumption. For the curve-fitting, we employed following equation:

$$\delta_i = \delta_\alpha - (\delta_\alpha - \delta_\xi)\left(1 + \frac{1 - \sqrt{4c_T K_E + 1}}{2c_T K_E}\right)$$

where δ_i and c_T are observable values of the chemical shift and total concentration, δ_α, δ_ξ and K_a are fitting parameters denote chemical shift of monomer, chemical shift of molecules in the assembly and the association constant, respectively. The mass fractions of monomer and n-mer, α and α_n, were calculated by using following equations:

$$\alpha = \frac{2c_T K_E + 1 - \sqrt{4c_T K_E + 1}}{2{c_T}^2 {K_E}^2}$$

$$\alpha_n = n a^n {c_T}^{n-1} {K_E}^{n-1}$$

2. ^{1}H NMR of DPA[n] at different concentration

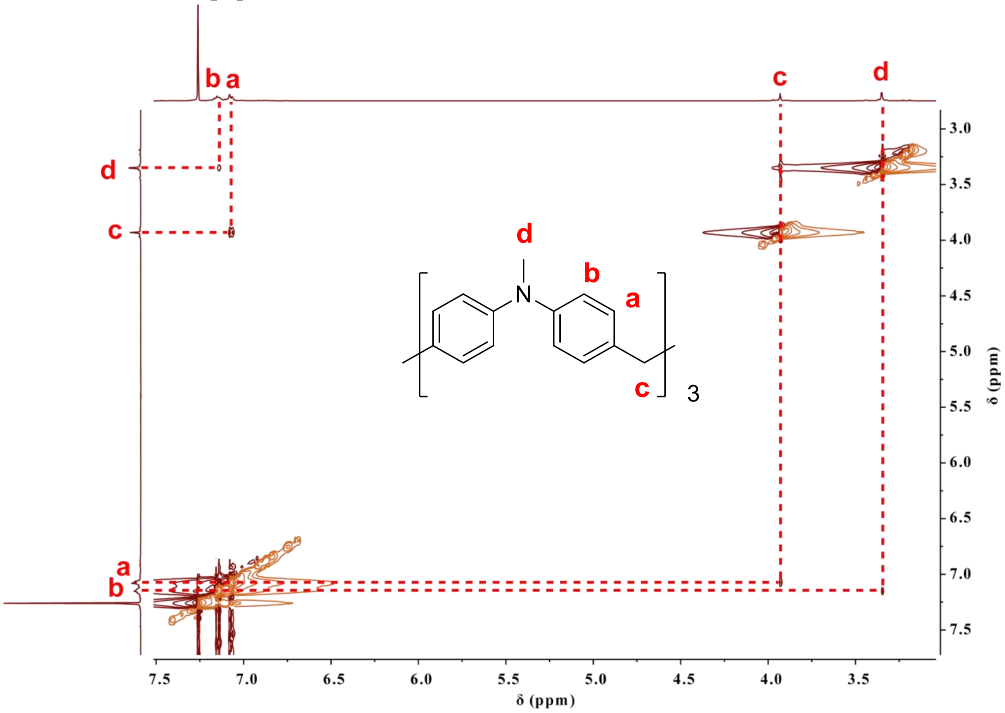

Supplementary Figure 7. 2D NOESY (500 MHz, 298 K,) of **DPA[3]** (2.0 mM) in $CDCl_3$.

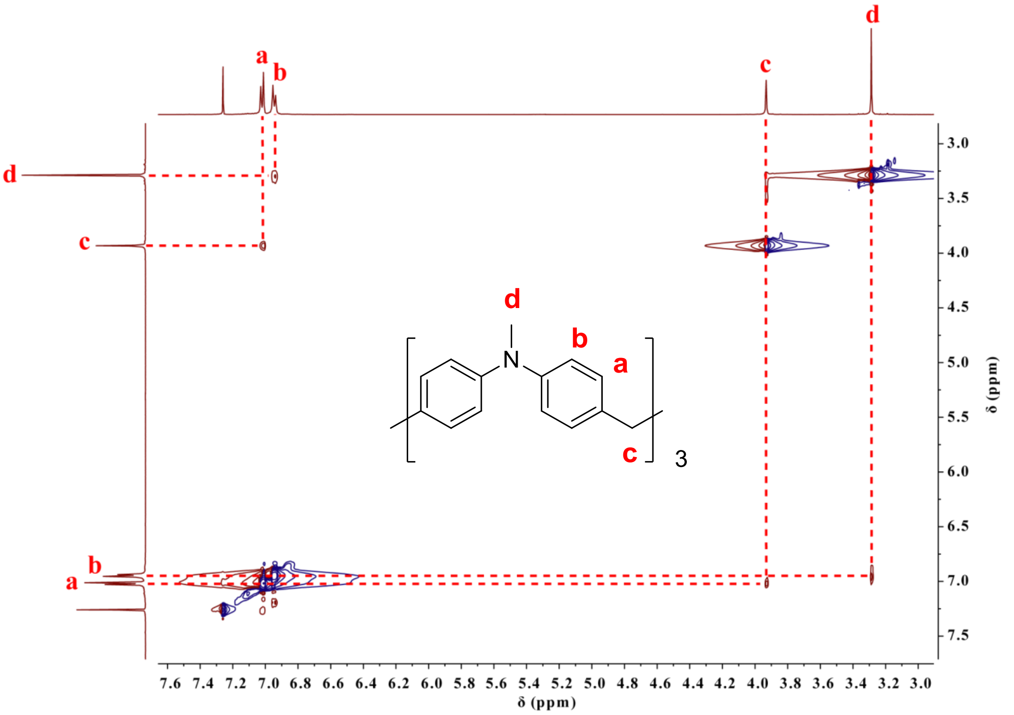

Supplementary Figure 8. 2D NOESY (500 MHz, 298 K) of **DPA[3]** (10 mM) in $CDCl_3$.

Concentration-dependent chemical shifts of **DPA[3]** aromatic resonances are shown in Supplementary Figure 9, and the results of curve-fitting analyses are shown in Supplementary Figure 10. Fitting parameters are summarized in Supplementary Table 4.

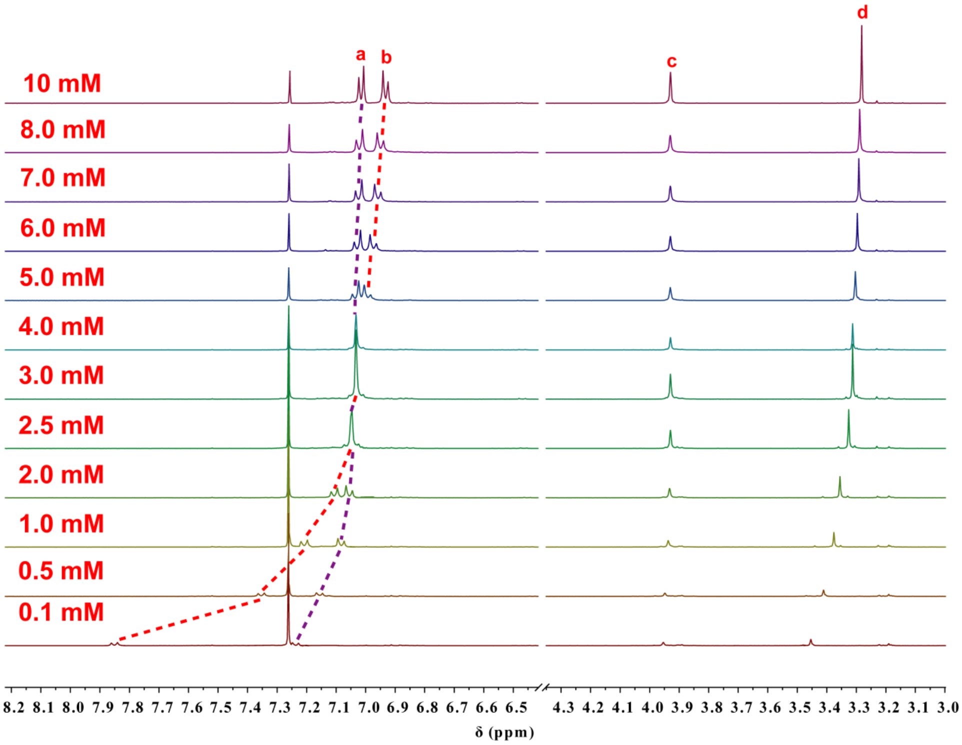

Supplementary Figure 9. Partial stacked ^{1}H NMR spectra (400 MHz, 298 K) of **DPA[3]** from 0.1 mM to 10 mM in $CDCl_3$.

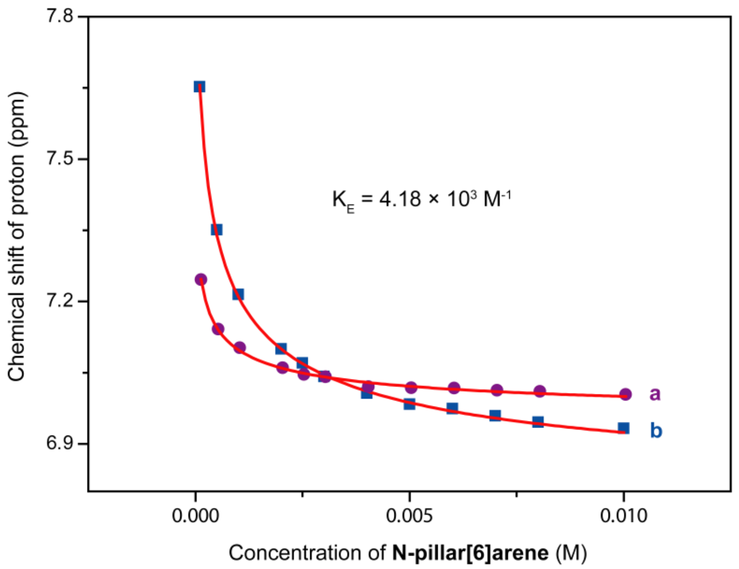

Supplementary Figure 10. Curve-fitting analysis of concentration-dependent chemical shifts of aromatic resonances of **DPA[3]**. The curves are derived from the fitting analysis and the association constants K_E were shown in the figure.

Supplementary Table 4. Calculated fitting parameters (association constant (K_E) chemical shifts of monomer (δ_α) and molecule in the assembly (δ_ξ) and R^2 values at 298 K for **DPA[3]**.

proton	K_E (M^{-1})	δ_α	δ_ξ	R^2
a	4.20×10^3	7.354	6.953	0.994
b	4.15×10^3	7.940	6.753	0.999
average	4.18×10^3			

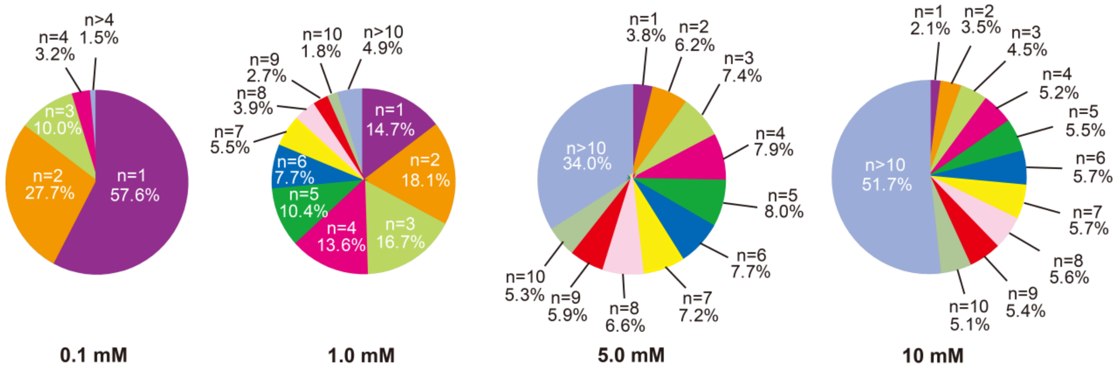

Supplementary Figure 11. Populations of oligomeric n-mers in terms of mass fractions of **DPA[3]**.

Concentration-dependent chemical shifts of **DPA[4]** aromatic resonances are shown in Supplementary Figure 12, and the results of curve-fitting analyses are shown in Supplementary Figure 13. Fitting parameters are summarized in Supplementary Table 5.

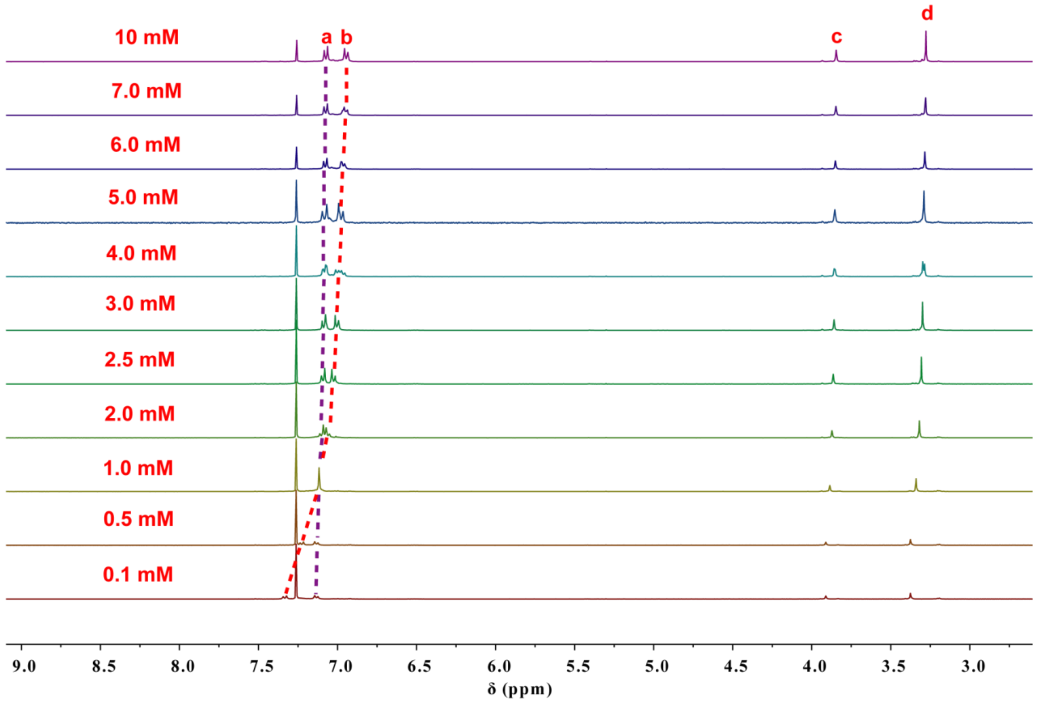

Supplementary Figure 12. Partial stacked ^{1}H NMR spectra (400 MHz, 298 K) of **DPA[4]** from 0.1 mM to 10 mM in $CDCl_3$.

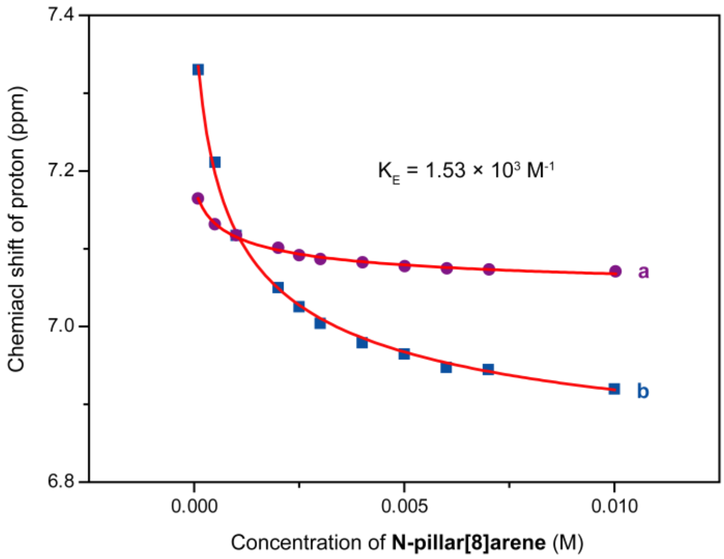

Supplementary Figure 13. Curve-fitting analysis of concentration-dependent chemical shifts of aromatic resonances of **DPA[4]**. The curves are derived from the fitting analysis and the association constants K_E were shown in the figure.

Supplementary Table 5. Calculated fitting parameters (association constant (K_E) chemical shifts of monomer (δ_α) and molecule in the assembly (δ_ξ) and R^2 values at 298 K for **DPA[4]**.

proton	K_E (M^{-1})	δ_α	δ_ξ	R^2
a	1.52×10^3	7.182	7.034	0.995
b	1.53×10^3	7.410	6.776	0.995
average	1.53×10^3			

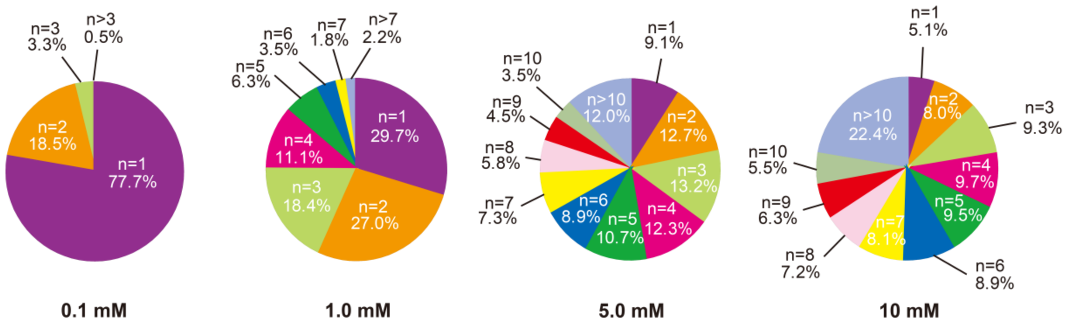

Supplementary Figure 14. Populations of oligomeric n-mers in terms of mass fractions of **DPA[4]**.

3. DOSY of DPA[n]

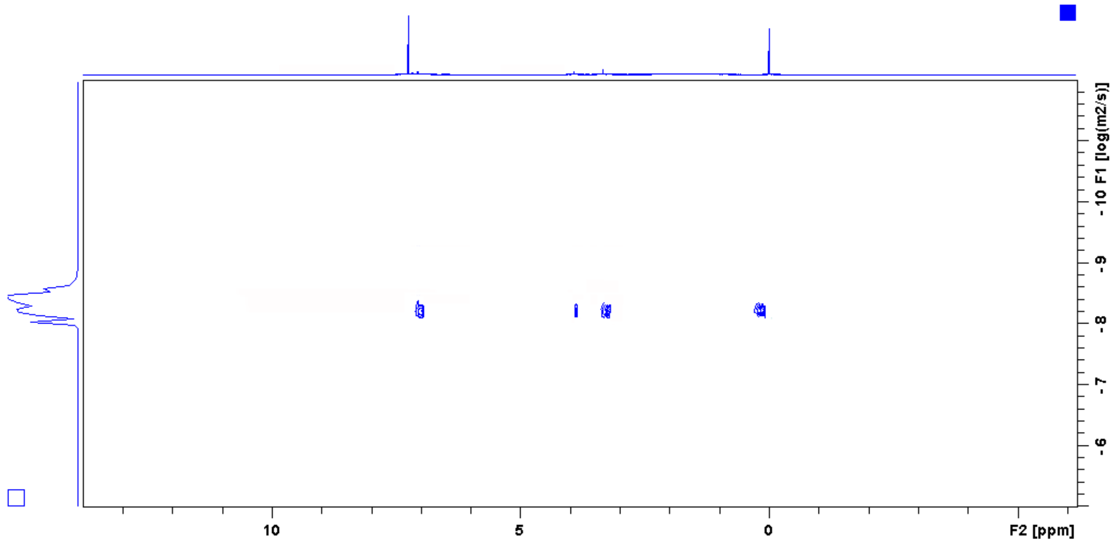

Supplementary Figure 15. DOSY (500 MHz, 298 K) of 1.0 mM **DPA[3]** in $CDCl_3$.

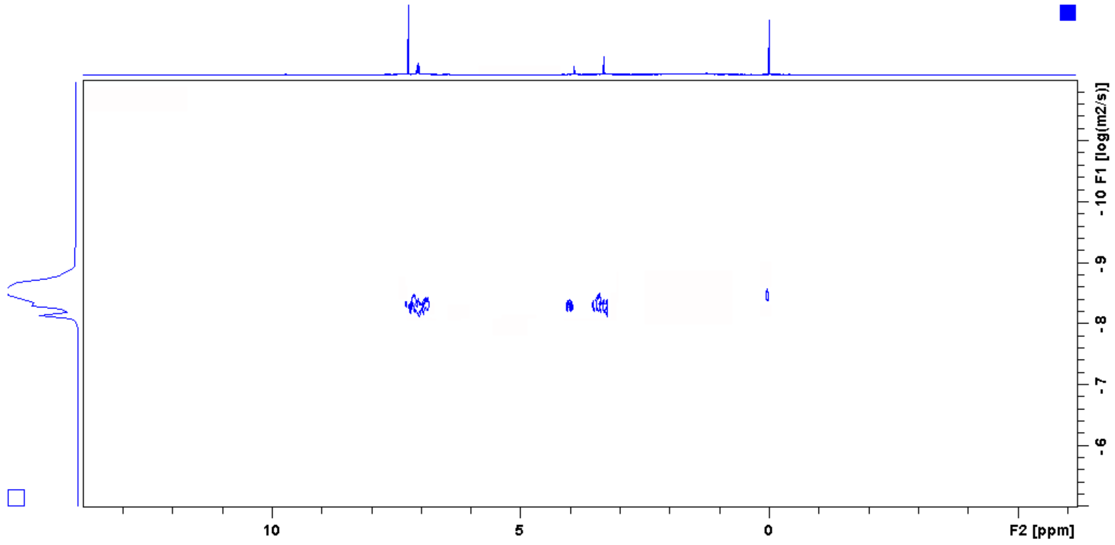

Supplementary Figure 16. DOSY (500 MHz, 298 K) of 2.0 mM **DPA[3]** in $CDCl_3$.

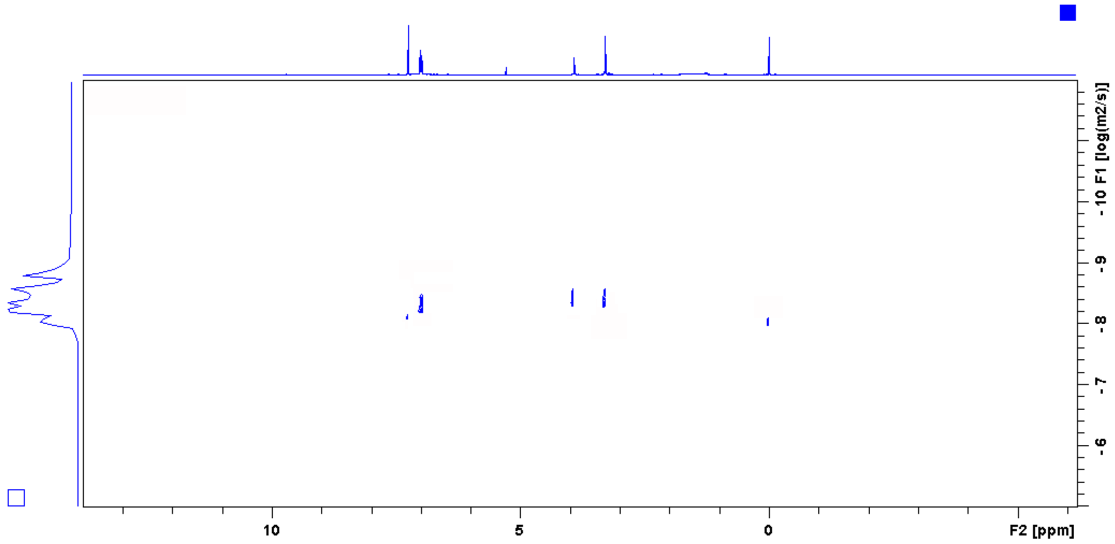

Supplementary Figure 17. DOSY (500 MHz, 298 K) of 5.0 mM **DPA[3]** in $CDCl_3$.

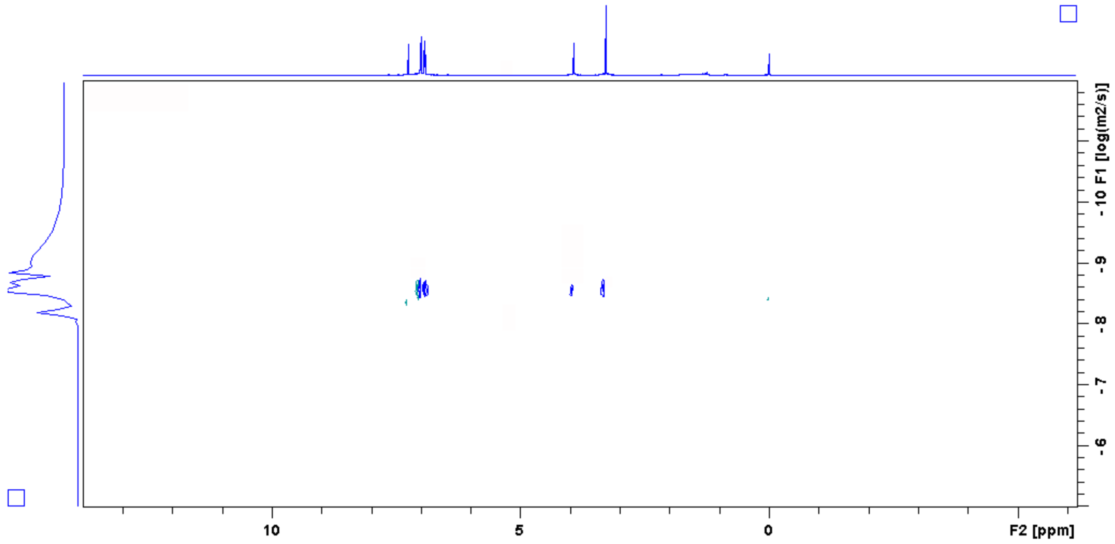

Supplementary Figure 18. DOSY (500 MHz, 298 K) of 10 mM **DPA[3]** in $CDCl_3$.

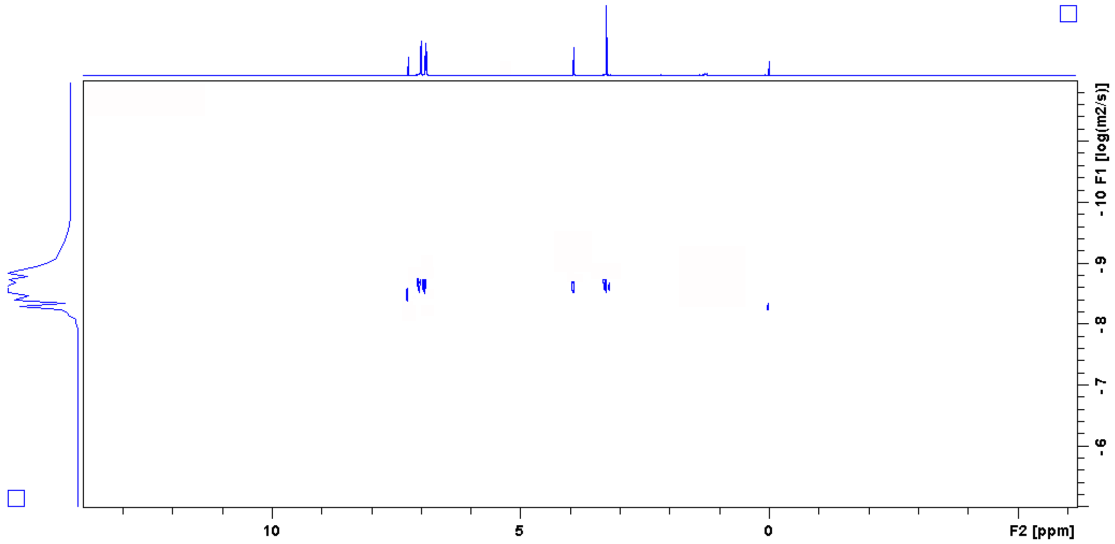

Supplementary Figure 19. DOSY (500 MHz, 298 K) of 20 mM **DPA[3]** in $CDCl_3$.

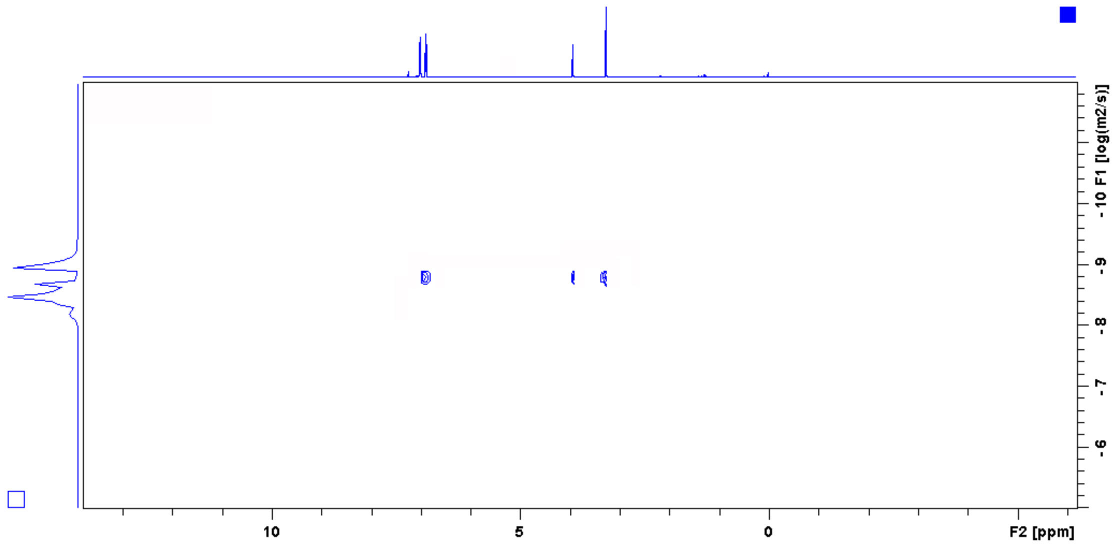

Supplementary Figure 20. DOSY (500 MHz, 298 K) of 50 mM **DPA[3]** in $CDCl_3$.

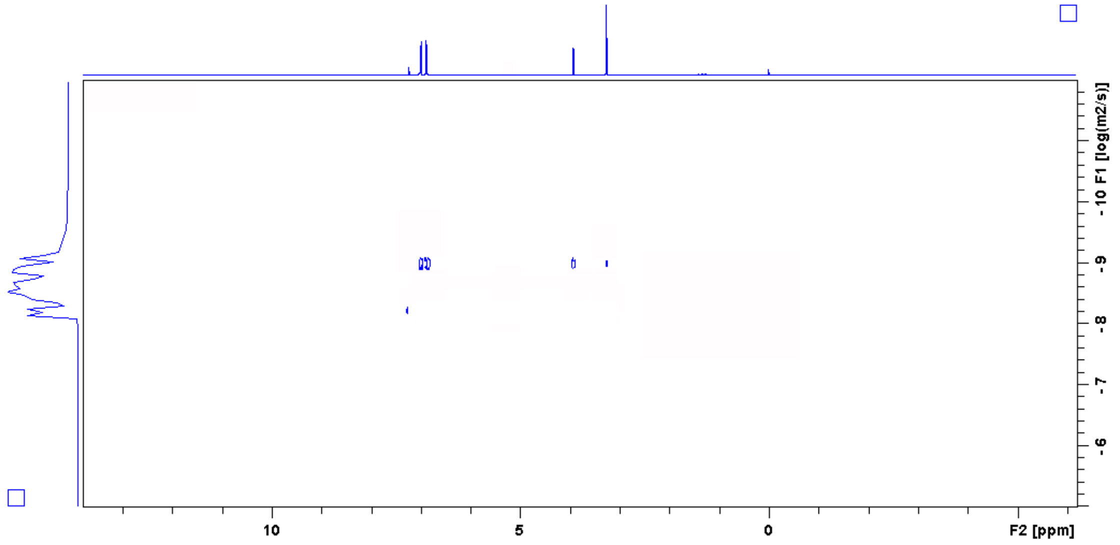

Supplementary Figure 21. DOSY (500 MHz, 298 K) of 100 mM **DPA[3]** in $CDCl_3$.

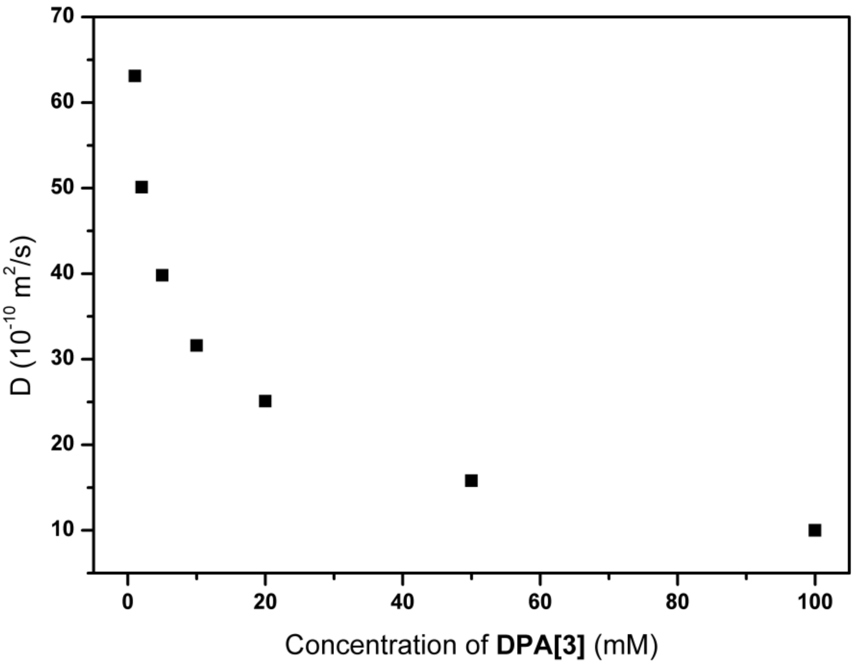

Supplementary Figure 22. Concentration dependence of diffusion coefficient D (500 MHz, 298 K) in $CDCl_3$ of **DPA[3]**.

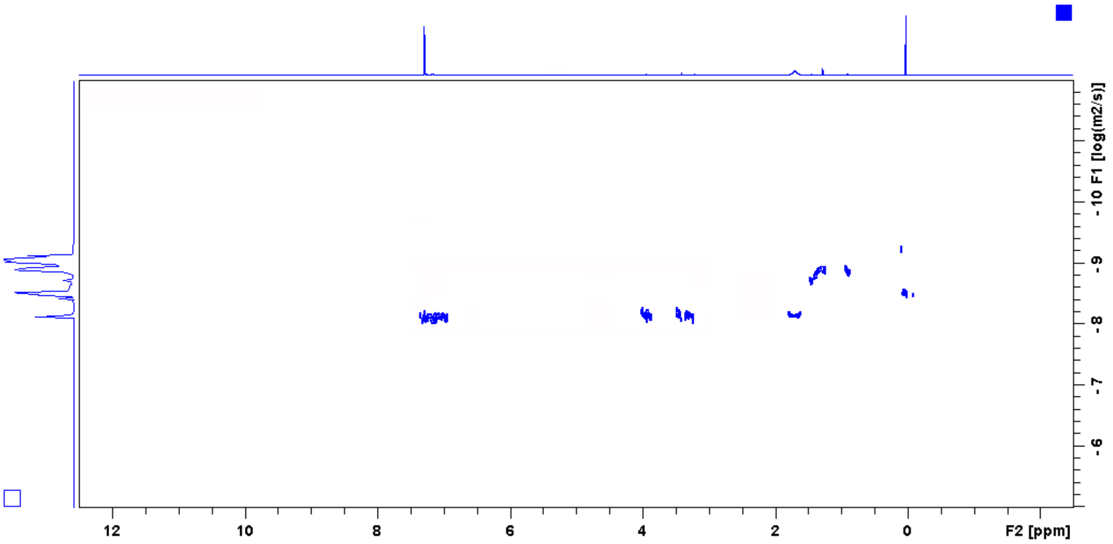

Supplementary Figure 23. DOSY (500 MHz, 298 K) of 1.0 mM **DPA[4]** in $CDCl_3$.

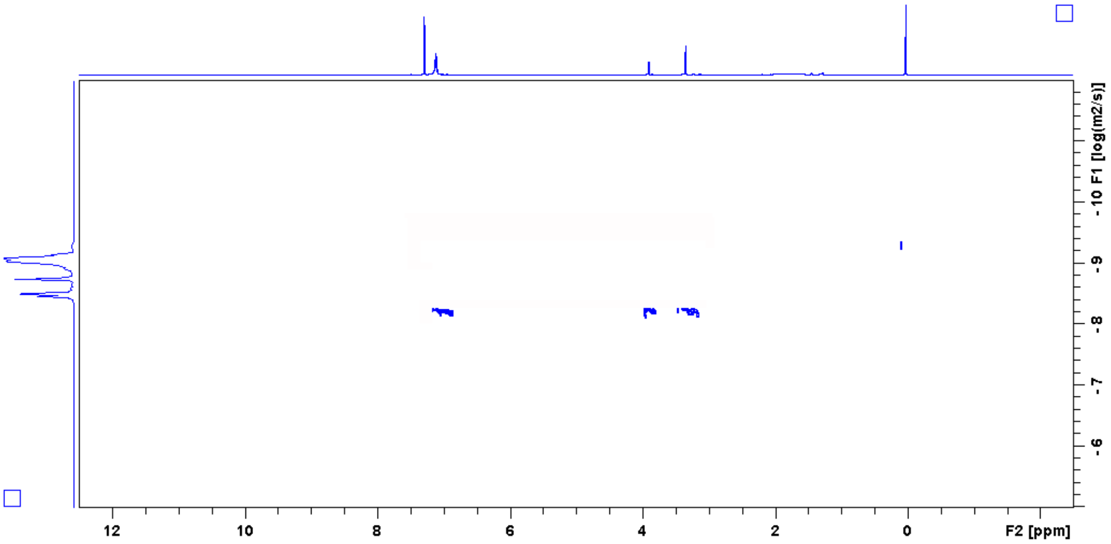

Supplementary Figure 24. DOSY (500 MHz, 298 K) of 2.0 mM **DPA[4]** in $CDCl_3$.

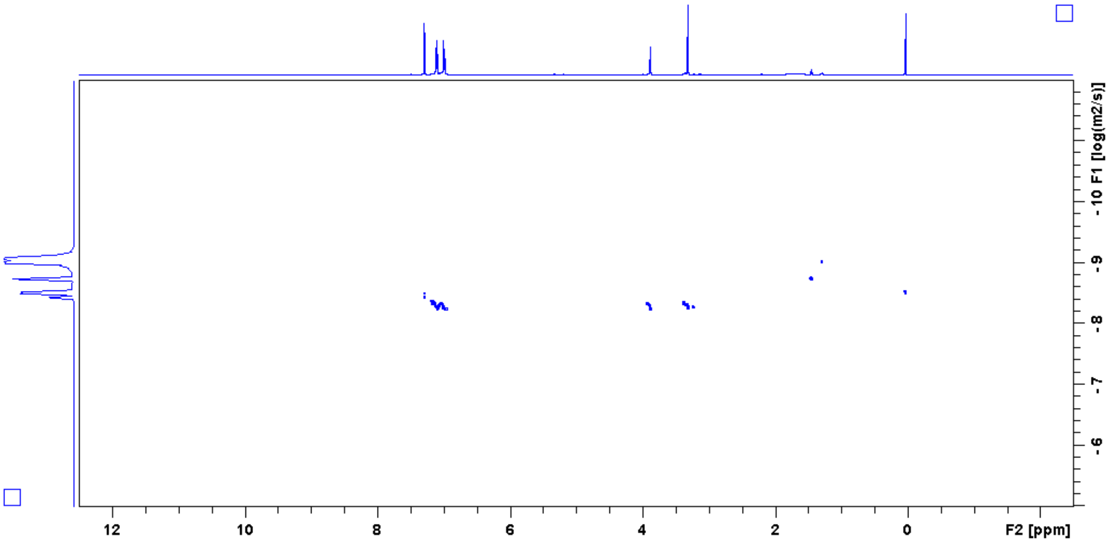

Supplementary Figure 25. DOSY (500 MHz, 298 K) of 5.0 mM **DPA[4]** in $CDCl_3$.

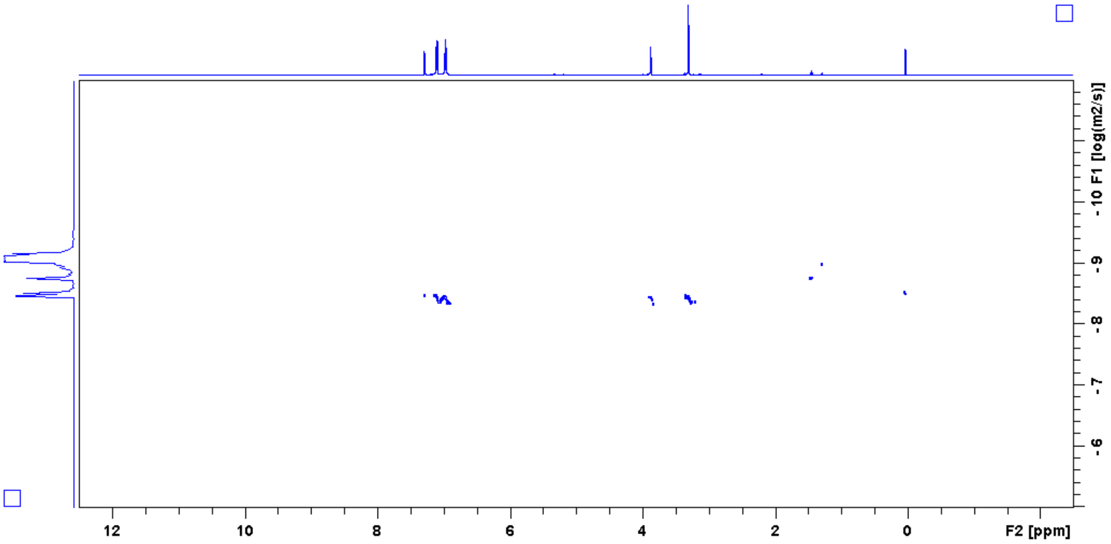

Supplementary Figure 26. DOSY (500 MHz, 298 K) of 10 mM **DPA[4]** in $CDCl_3$.

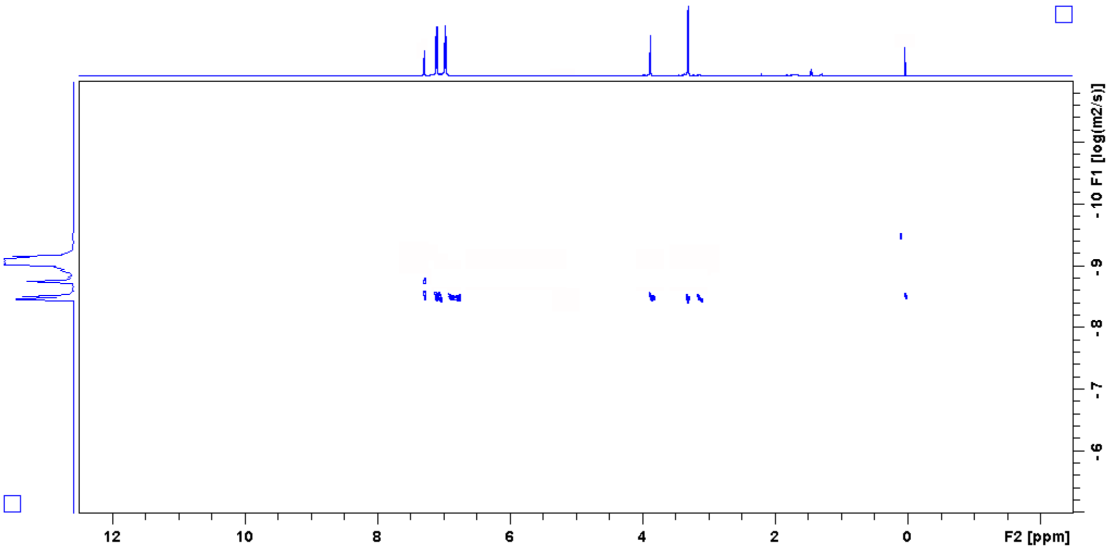

Supplementary Figure 27. DOSY (500 MHz, 298 K) of 20 mM **DPA[4]** in $CDCl_3$.

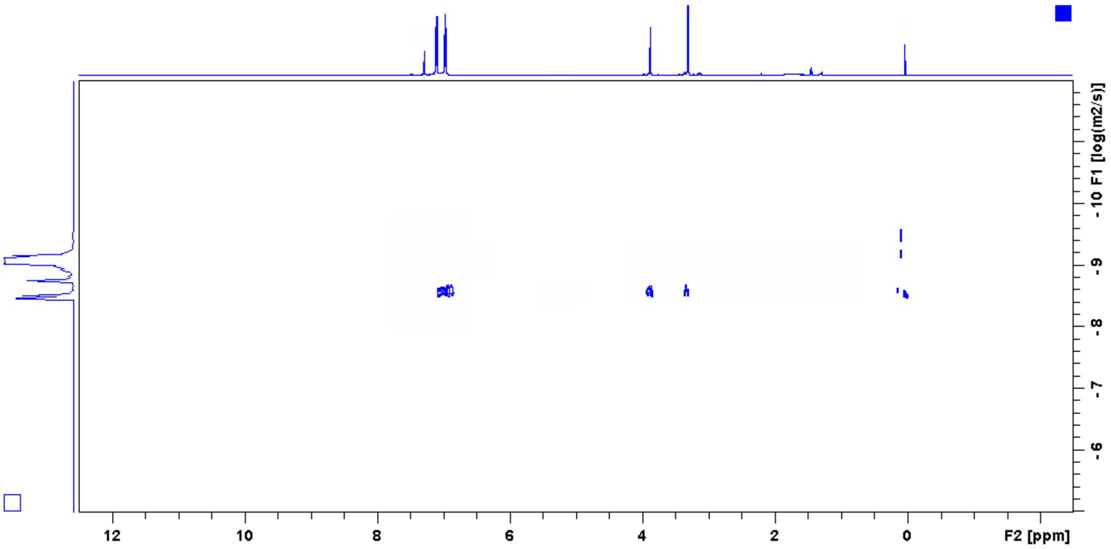

Supplementary Figure 28. DOSY (500 MHz, 298 K) of 50 mM **DPA[4]** in $CDCl_3$.

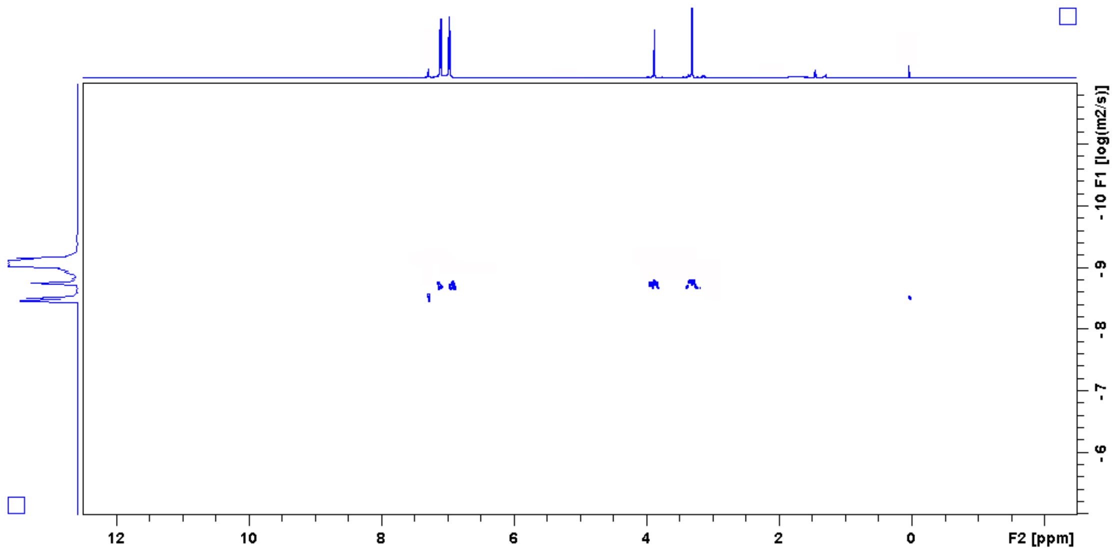

Supplementary Figure 29. DOSY (500 MHz, 298 K) of 100 mM **DPA[4]** in $CDCl_3$.

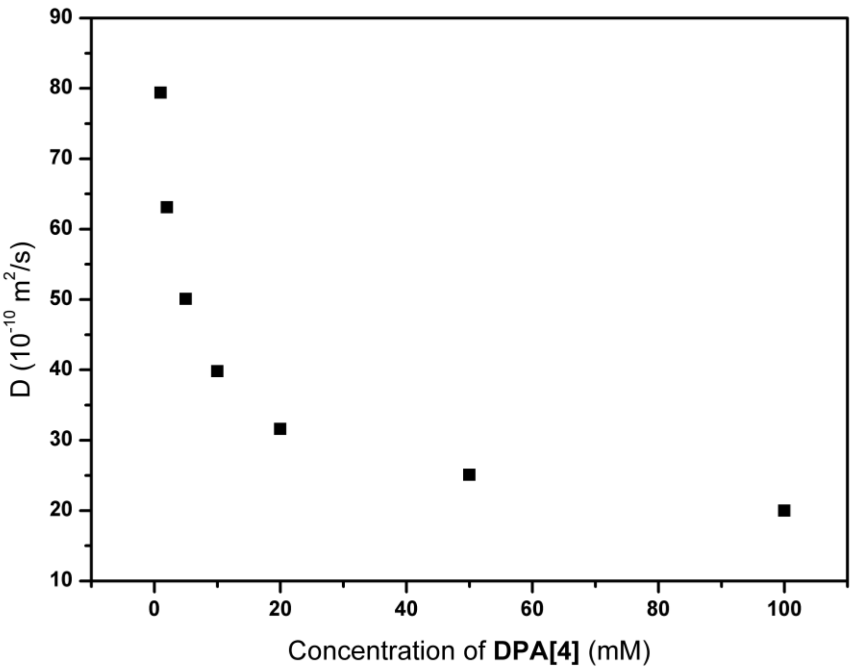

Supplementary Figure 30. Concentration dependence of diffusion coefficient D (500 MHz, 298 K) in $CDCl_3$ of **DPA[4]**.

4. AFM image of DPA[n]

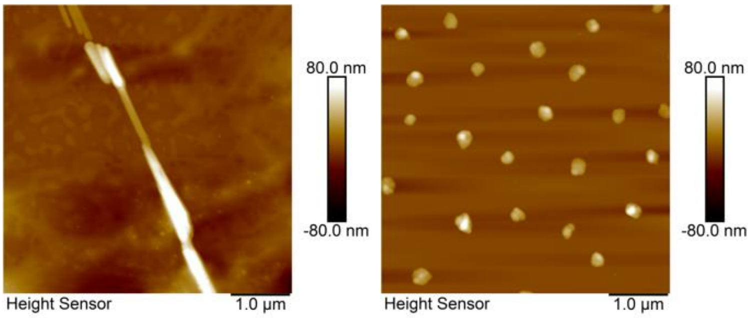

Supplementary Figure 31. AFM images of **DPA[3]** at high concentration (1.0 mM, left) and low concentration (0.2 mM, right).

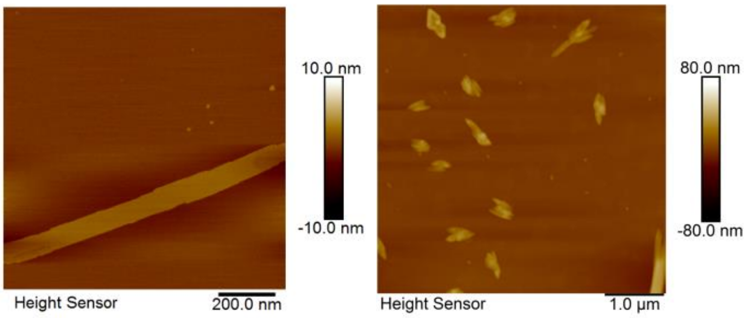

Supplementary Figure 32. AFM images of **DPA[4]** at high concentration (1.0 mM, left) and low concentration (0.2 mM, right).

4. SEM image of DPA[n]

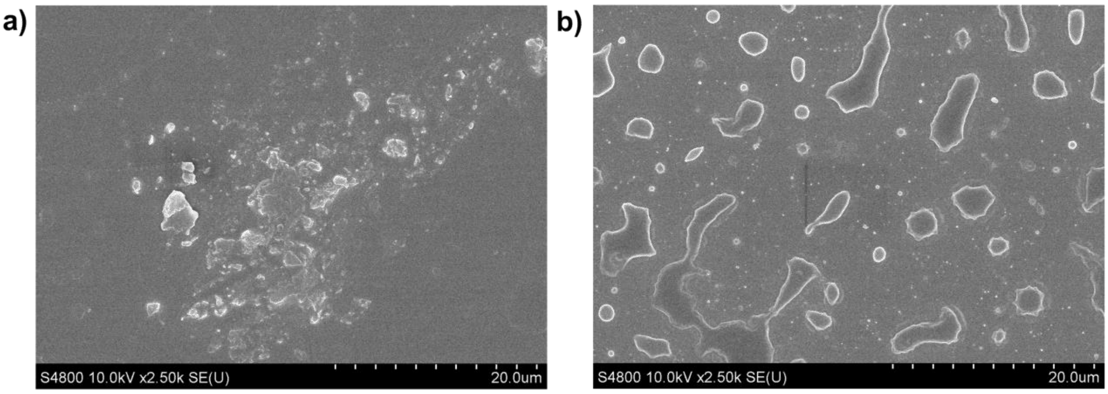

Supplementary Figure 33. SEM images of **DPA[3]** (a) and **DPA[4]** (b) at a low concentration (0.1 mM).

Section F. Host-guest interaction

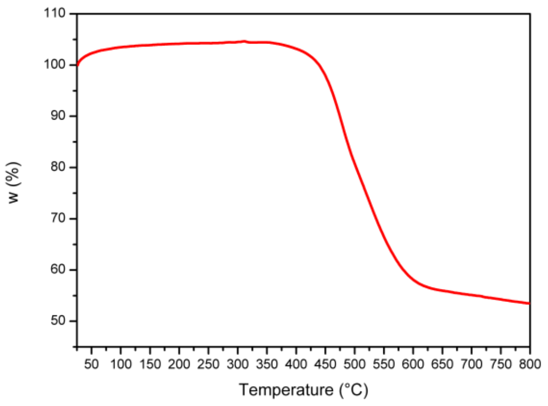

Supplementary Figure 34. TGA curve of **DPA[3]** powder.

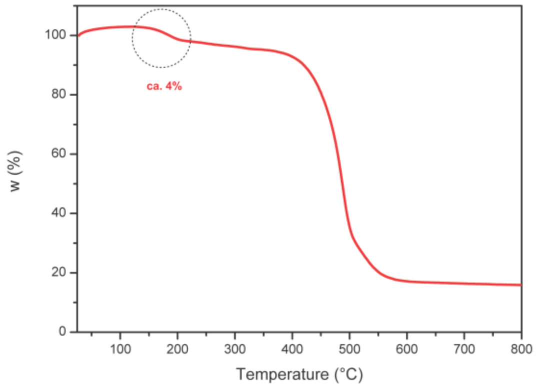

Supplementary Figure 35. TGA curve of **DPA[3]** powder immersed in ethene atmosphere for 48 h.

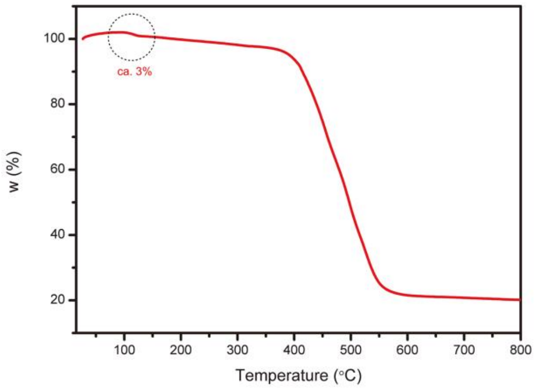

Supplementary Figure 36. TGA curve of **DPA[3]** powder immersed in ethyne atmosphere for 48 h.

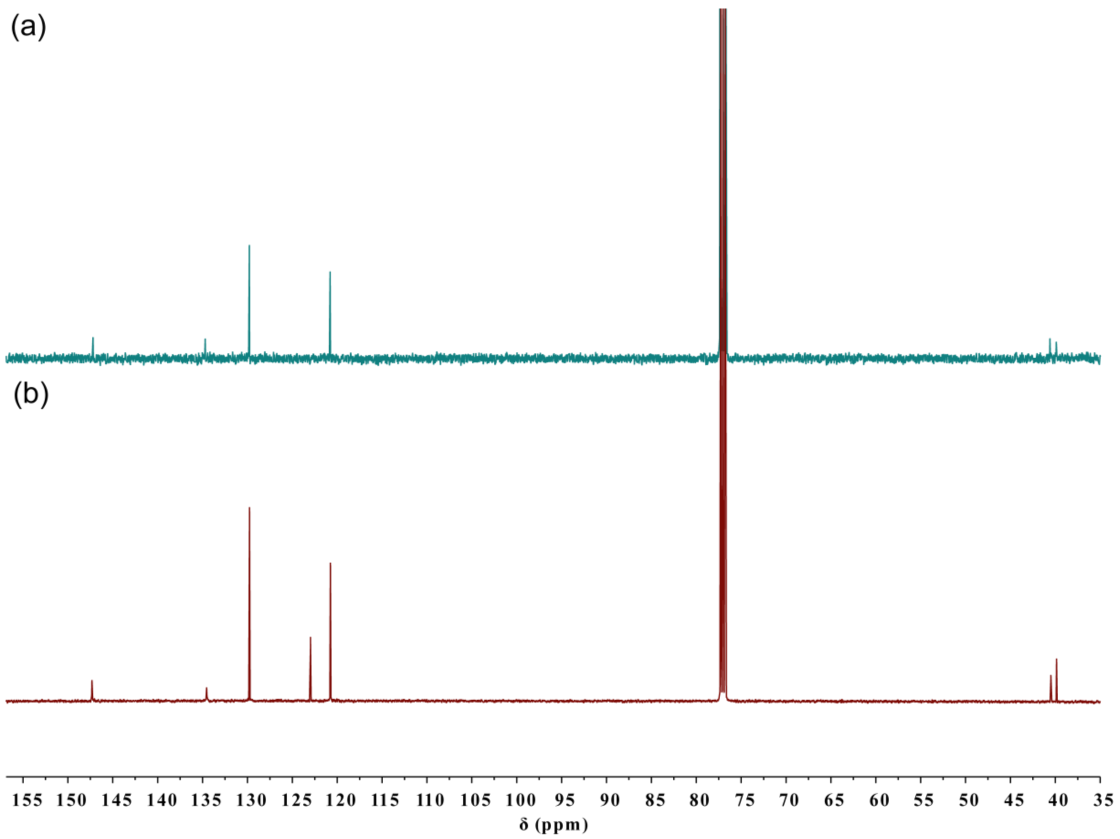

Supplementary Figure 37. Partial stacked ^{13}C NMR spectra (100 MHz, 298 K) of **DPA[3]** (a) and **DPA[3]** powder immersed in ethene atmosphere for 48 h (b) in $CDCl_3$.

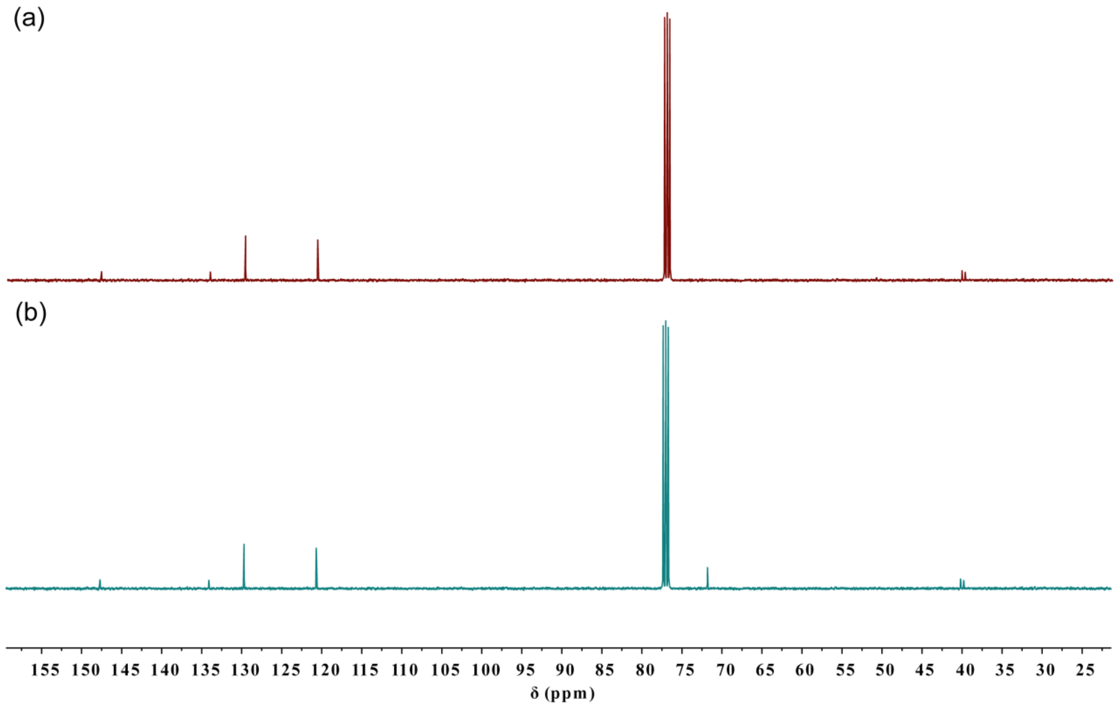

Supplementary Figure 38. Partial stacked ^{13}C NMR spectra (100 MHz, 298 K) of **DPA[3]** (a) and **DPA[3]** powder immersed in ethyne atmosphere for 48 h (b) in $CDCl_3$.

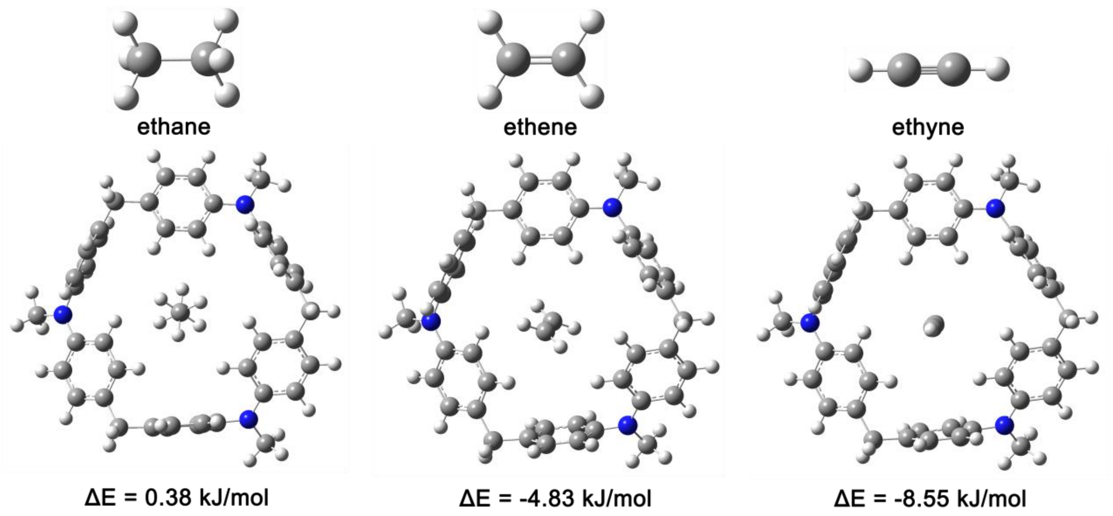

Supplementary Figure 39. Binding energies of host-guest complexes between **DPA[3]** and ethane, ethene, and ethyne obtained from DFT calculation based on B3LYP/6-31G level.

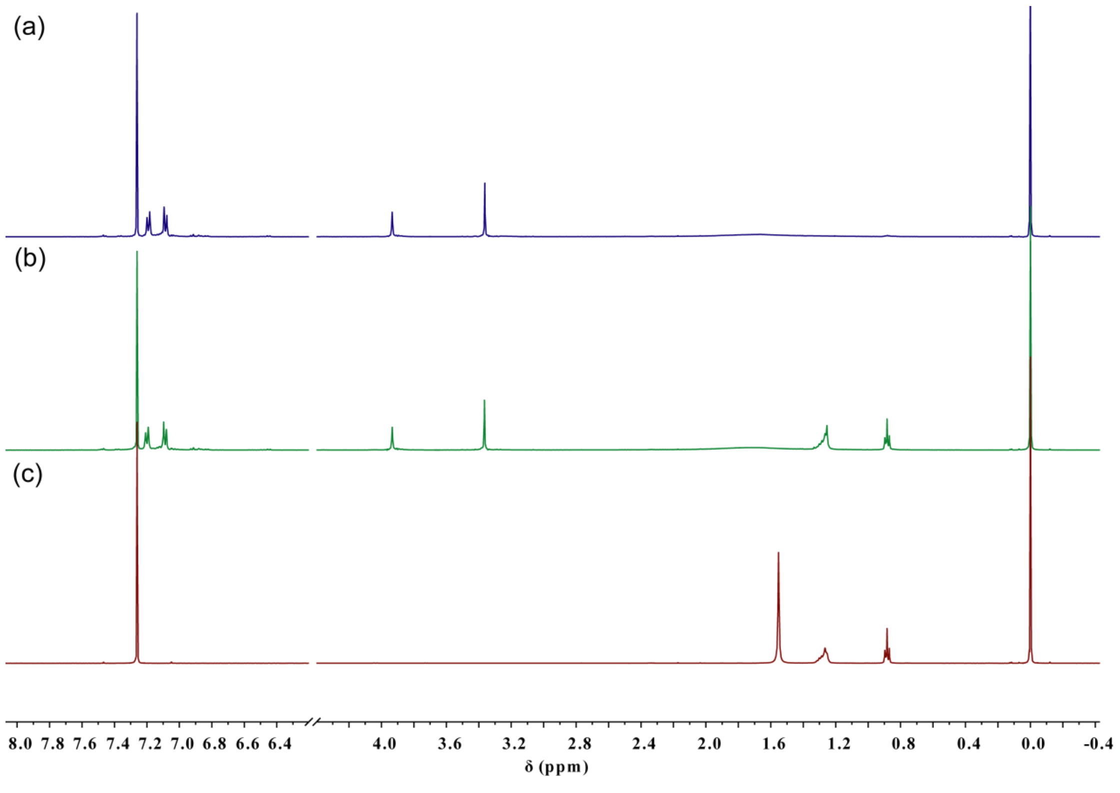

Supplementary Figure 40. Partial ^{1}H NMR spectra (400 MHz, $CDCl_3$, 298 K) of solutions of (a) 2.0 mM **DPA[3]**; (b) 2.0 mM **DPA[3]** and 4.0 mM *n*-hexane; and (c) 4.0 mM *n*-hexane.

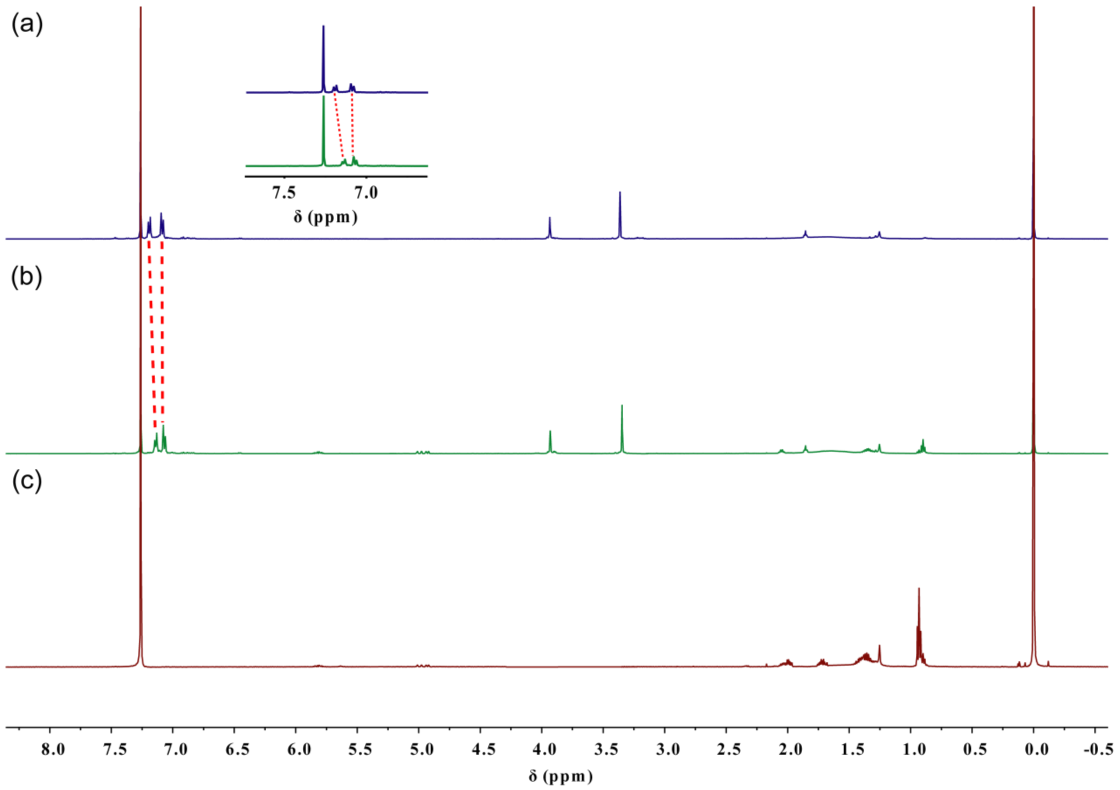

Supplementary Figure 41. Partial ^{1}H NMR spectra (400 MHz, $CDCl_3$, 298 K) of solutions of (a) 2.0 mM **DPA[3]**; (b) 2.0 mM **DPA[3]** and 4.0 mM *n*-hexylene; and (c) 4.0 mM *n*-hexylene.

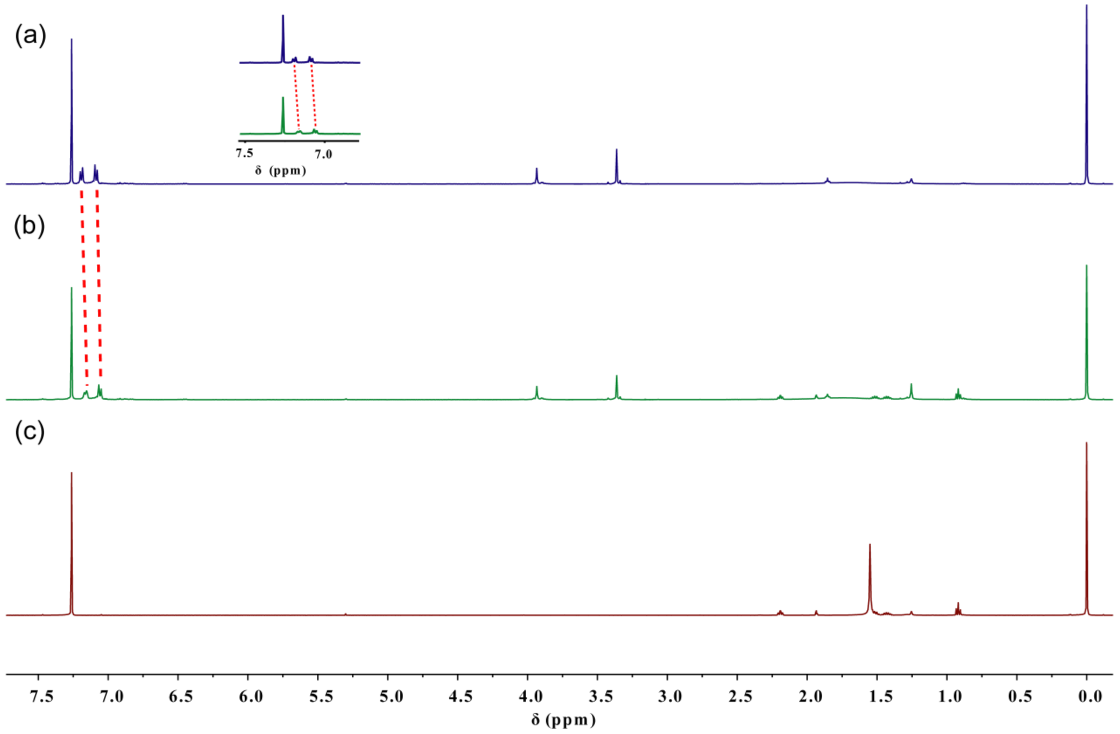

Supplementary Figure 42. Partial ^{1}H NMR spectra (400 MHz, $CDCl_3$, 298 K) of solutions of (a) 2.0 mM **DPA[3]**; (b) 2.0 mM **DPA[3]** and 4.0 mM *n*-hexyne; and (c) 4.0 mM *n*-hexyne.

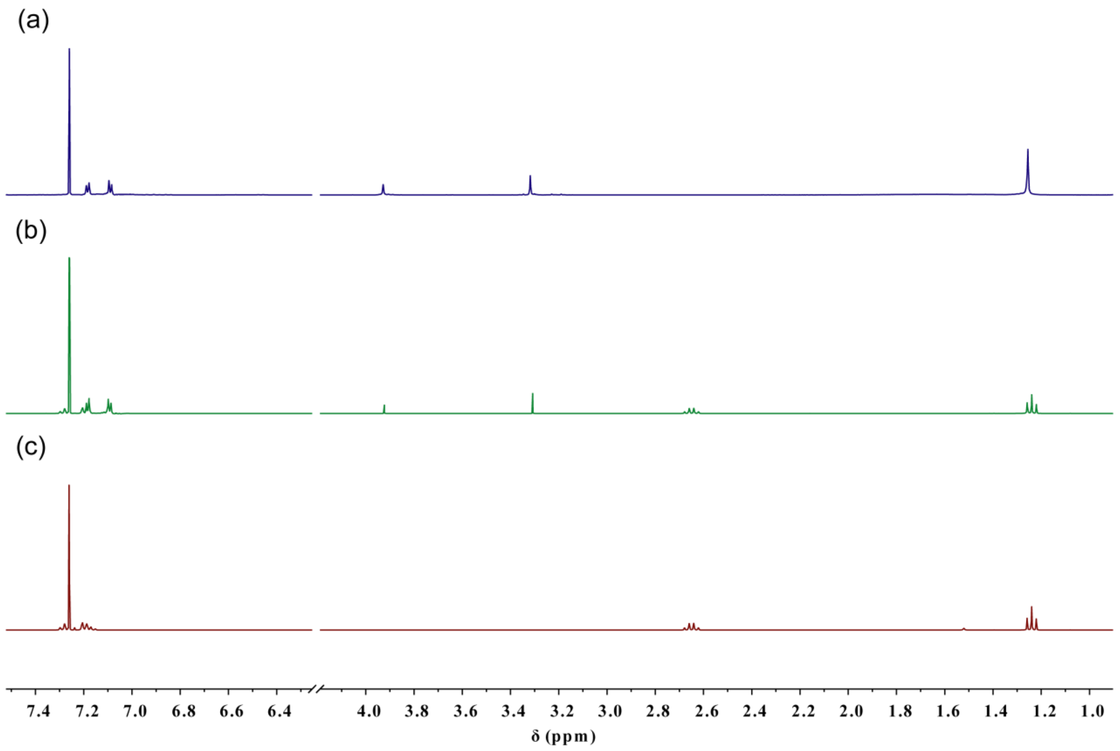

Supplementary Figure 43. Partial ^{1}H NMR spectra (400 MHz, $CDCl_3$, 298 K) of solutions of (a) 2.0 mM **DPA[3]**; (b) 2.0 mM **DPA[3]** and 4.0 mM ethylbenzene; and (c) 4.0 mM ethylbenzene.

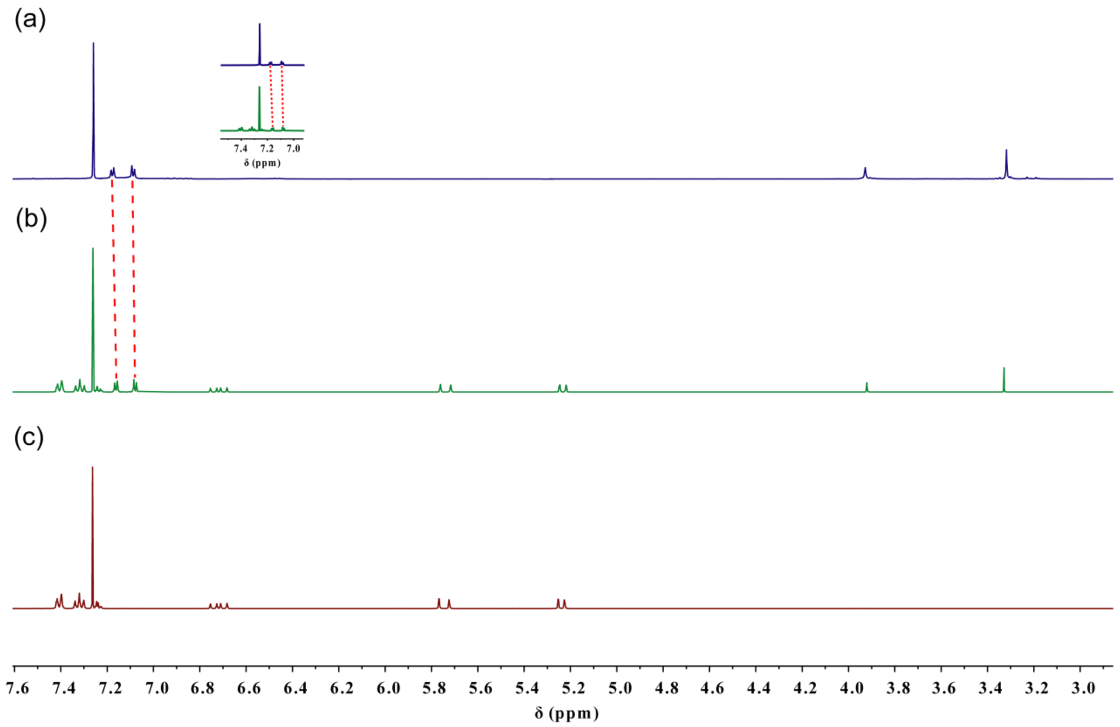

Supplementary Figure 44. Partial ^{1}H NMR spectra (400 MHz, $CDCl_3$, 298 K) of solutions of (a) 2.0 mM **DPA[3]**; (b) 2.0 mM **DPA[3]** and 4.0 mM styrene; and (c) 4.0 mM styrene.

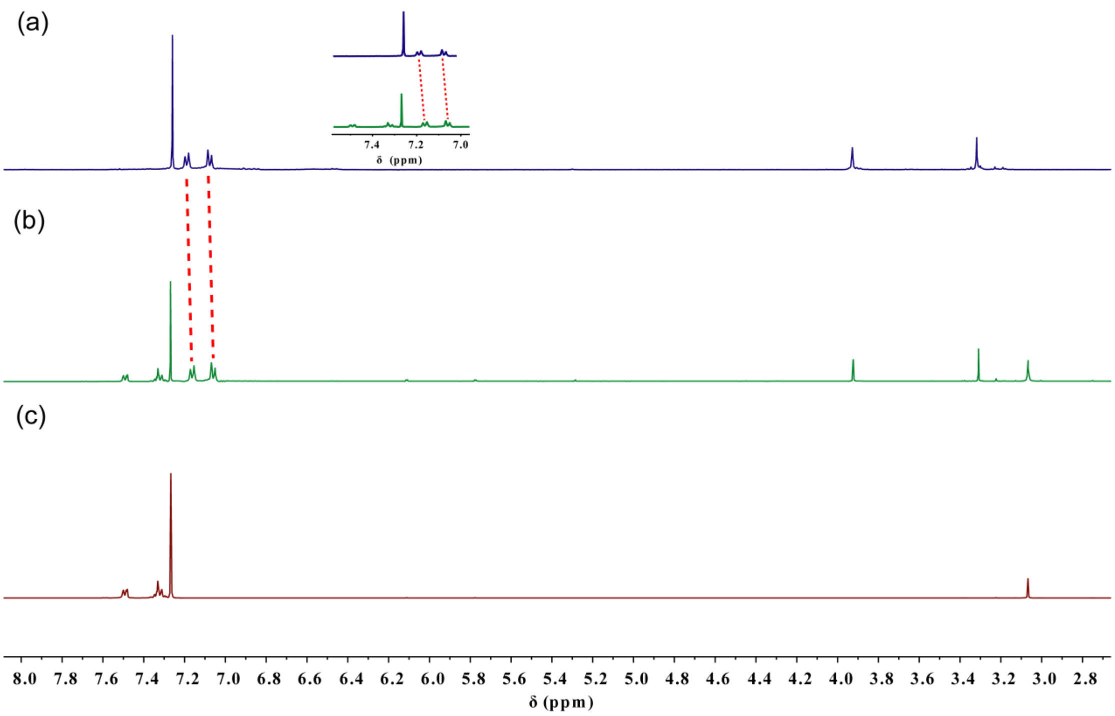

Supplementary Figure 45. Partial ^{1}H NMR spectra (400 MHz, $CDCl_3$, 298 K) of solutions of (a) 2.0 mM **DPA[3]**; (b) 2.0 mM **DPA[3]** and 4.0 mM phenylacetylene; and (c) 4.0 mM phenylacetylene.

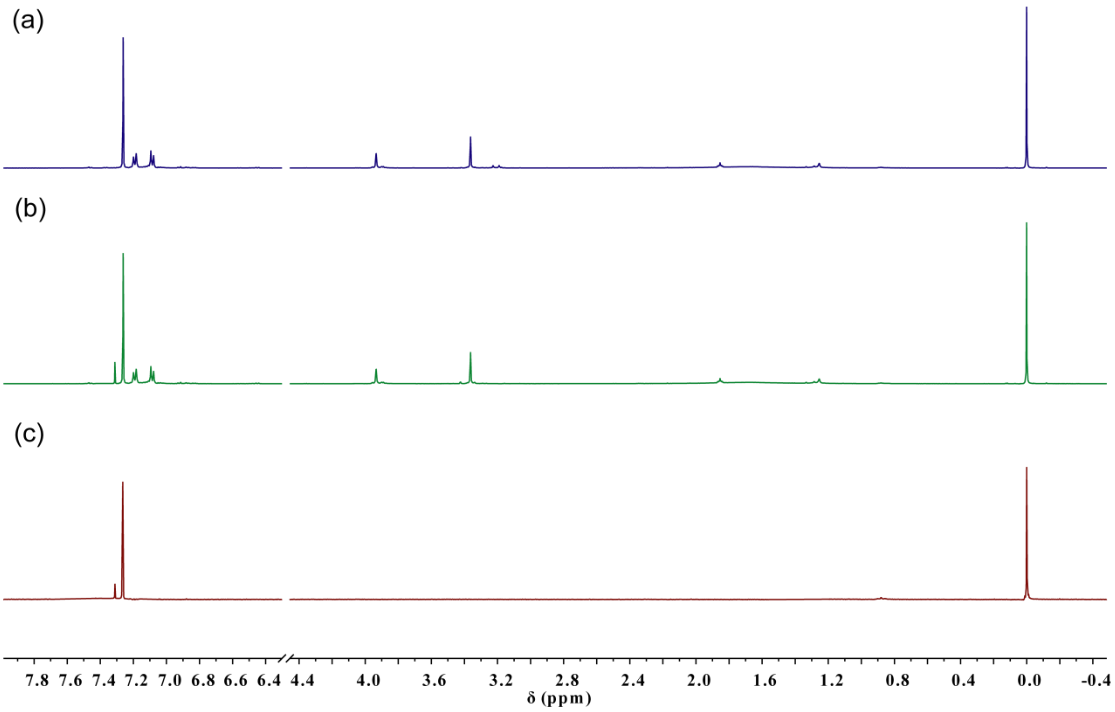

Supplementary Figure 46. Partial ^{1}H NMR spectra (400 MHz, $CDCl_3$, 298 K) of solutions of (a) 2.0 mM **DPA[3]**; (b) 2.0 mM **DPA[3]** and 4.0 mM benzene; and (c) 4.0 mM benzene.

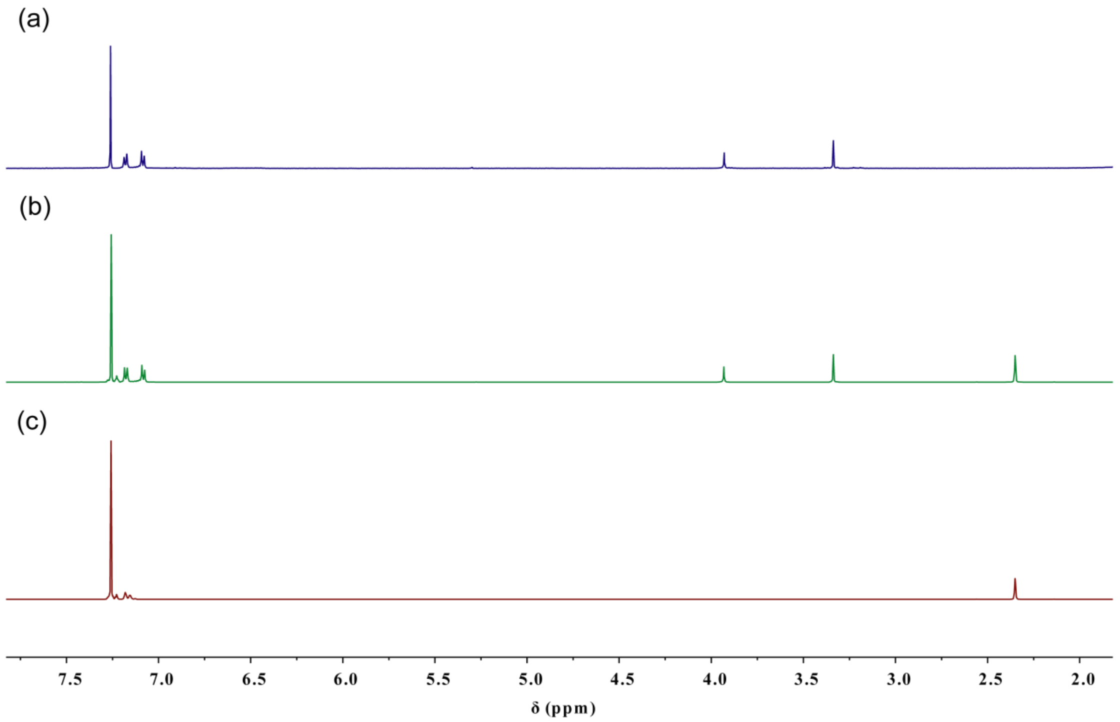

Supplementary Figure 47. Partial ^{1}H NMR spectra (400 MHz, $CDCl_3$, 298 K) of solutions of (a) 2.0 mM **DPA[3]**; (b) 2.0 mM **DPA[3]** and 4.0 mM toluene; and (c) 4.0 mM toluene.

Section G. X-ray crystal data

1. X-ray crystal data of DPA[3]

X-ray crystallography of single crystal obtained by vapor diffusion of MeOH (2 mL) into solutions of **DPA[3]** (2 mg) in $CHCl_3$ (1 mL). CCDC number: 1882113.

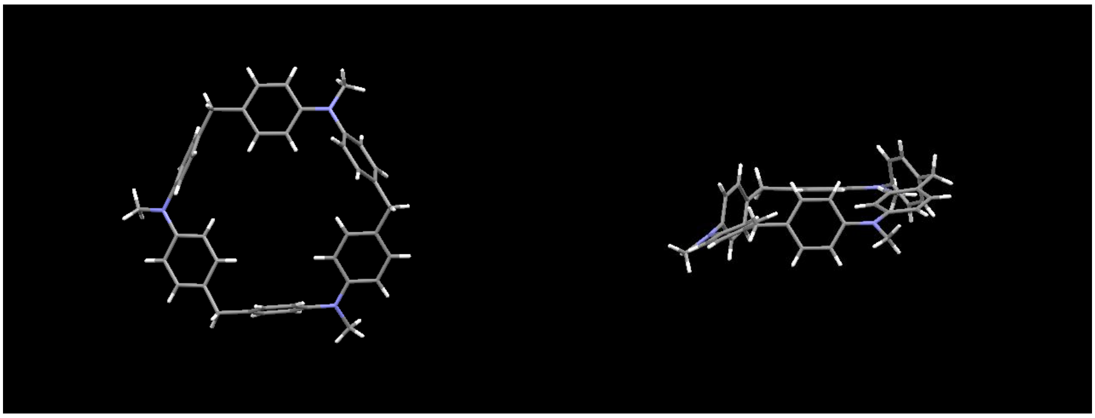

Supplementary Figure 48. X-ray crystal structure of **DPA[3]** with top and side views.

Supplementary Table 6. Crystal data and structure refinement parameters for **DPA[3]**.

Identification code	exp_566
Empirical formula	$C_{42}H_{39}N_3$
Formula weight	585.78
Temperature/K	100.01

Crystal system	trigonal
space group	R-3
Unit cell dimensions	a = 24.5908 Å α = 90 deg.
	b = 24.5908 Å β = 90 deg.
	c = 10.1302 Å γ = 120 deg.
Volume/$Å^3$	5296.6
Z	18
$\rho_{calc}g/cm^3$	1.102
Absorption coefficient/mm^{-1}	0.489
F(000)	1872.0
Crystal size/mm^3	0.48 × 0.26 × 0.12
Radiation	CuKα (λ = 1.54184)
2θ range for data collection/°	7.19 to 153.31
Index ranges	$-30 \le h \le 30$
	$-29 \le k \le 30$
	$-12 \le l \le 12$
Reflections collected	26230
Independent reflections	2392 [R_{int} = 0.2015, R_{sigma} = 0.0599]
Data/restraints/parameters	2392/0/138
Goodness-of-fit on F^2	1.060
Final R indexes [I>=2σ (I)]	R_1 = 0.0891, wR_2 = 0.2334
Final R indexes [all data]	R_1 = 0.1004, wR_2 = 0.2411
Largest diff. peak/hole / e $Å^{-3}$	1.03/-0.32

2. X-ray crystal data of DPA[4]

X-ray crystallography of single crystal obtained by vapor diffusion of MeOH (2 mL) into solutions of **DPA[4]** (2 mg) in $CHCl_3$ (1 mL). CCDC number: 1948482.

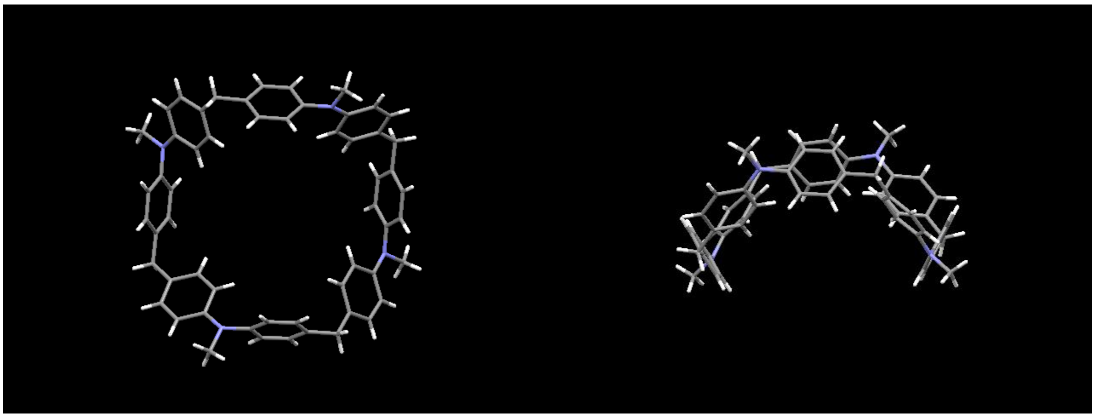

Supplementary Figure 49. X-ray crystal structure of **DPA[4]** with top and side views.

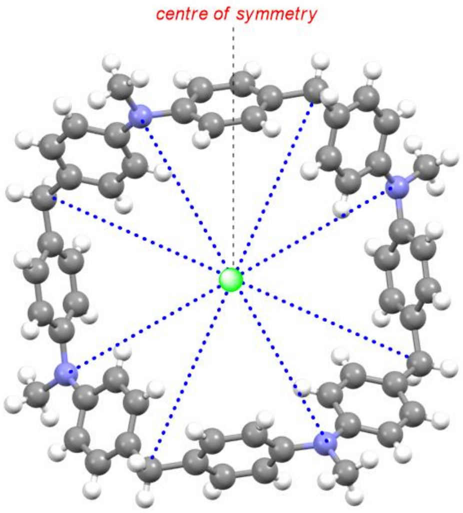

Supplementary Figure 50. The symmetry centre of **DPA[4]**.

Supplementary Table 7. Crystal data and structure refinement parameters for **DPA[4]**.

Identification code	exp_672
Empirical formula	$C_{56}H_{52}N_4$
Formula weight	781.04
Temperature/K	100.00
Crystal system	orthorhombic
Space group	$P2_12_12$
Unit cell dimensions	a = 21.5460 Å α = 90 deg.
	b = 21.9385 Å β = 90 deg.
	c = 5.5463 Å γ = 90 deg.
Volume/Å^3	2621.7
Z	4
ρ_{calc}g/cm^3	0.989
Absorption coefficient/mm^{-1}	0.439
F(000)	832.0
Crystal size/mm^3	0.42 × 0.12 × 0.11
Radiation	CuKα (λ = 1.54184)
2θ range for data collection/°	8.208 to 157.866
Index ranges	$-26 \leq h \leq 26$
	$-27 \leq k \leq 27$
	$-6 \leq l \leq 7$
Reflections collected	71182
Independent reflections	5323 [R_{int} = 0.1273, R_{sigma} = 0.0511]
Data/restraints/parameters	5323/0/274
Goodness-of-fit on F^2	0.939
Final R indexes [I>=2σ (I)]	R_1 = 0.0554, wR_2 = 0.1293
Final R indexes [all data]	R_1 = 0.0692, wR_2 = 0.1364
Largest diff. peak/hole / e Å^{-3}	0.15/-0.19

Flack parameter	-0.4(4)

3. X-ray crystal data of DPA[3] ⊃ ethene

X-ray crystallography of single crystal obtained by immersing crystal of **DPA[3]** in ethene atmosphere for 48 h. CCDC number: 1948483.

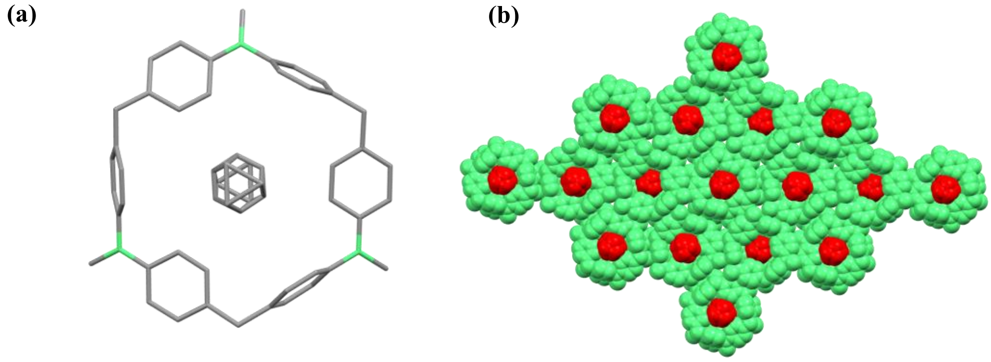

Supplementary Figure 51. X-ray crystal structure of **DPA[3]**⊃ethene.

Supplementary Table 8. Crystal data and structure refinement parameters for **DPA[3]**⊃ethene.

Identification code	exp_886
Empirical formula	$C_{44}H_{43}N_3$
Formula weight	613.83
Temperature/K	293
Crystal system	hexagonal
space group	R-3
Wavelength	1.54184 A
Unit cell dimensions	a = 24.5686 Å α = 90 deg.
	b = 24.5686 Å β = 90 deg.
	c = 10.1302 Å γ = 120 deg.
Volume/Å^3	5295.5
Z	23
Absorption coefficient/mm^{-1}	0.507
F(000)	1909
Crystal size/mm^3	0.26 × 0.12 × 0.11
Radiation	CuKα (λ = 1.54184)
2θ range for data collection/°	4.83 to 74.93 deg.
Index ranges	-30 ≤ h ≤ 30
	-30 ≤ k ≤ 30
	-12 ≤ l ≤ 12
Reflections collected	36371
Completeness to theta = 74.93	98.1 %
Max. and min. transmission	0.9463 and 0.8794
Refinement method	Full-matrix least-squares on F^2
Data / restraints / parameters	2390 / 14 / 173
Goodness-of-fit on F^2	1.064

Final R indices [I>2sigma(I)]	R1 = 0.0984, wR2 = 0.2764
R indices (all data)	R1 = 0.1120, wR2 = 0.2897
Extinction coefficient	0.0019(5)
Largest diff. peak and hole	0.538 and -0.545 e.A^-3

4. X-ray crystal data of DPA[3] ⊃ ethyne

X-ray crystallography of single crystal obtained by immersing crystal of **DPA[3]** in ethyne atmosphere for 48 h. CCDC number: 1975012.

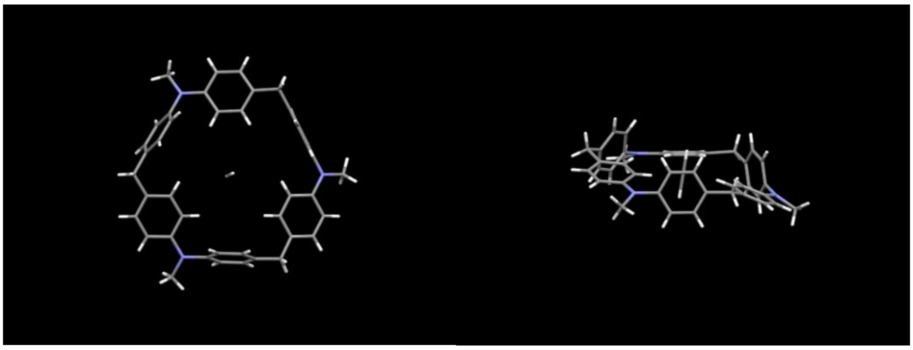

Supplementary Figure 52. X-ray crystal structure of **DPA[3]**⊃ethyne.

Supplementary Table 9. Crystal data and structure refinement parameters for **DPA[3]** ⊃ ethyne.

Identification code	sxl_sq
Empirical formula	$C_{44}H_{41}N_3$
Formula weight	611.82
Temperature/K	293
Crystal system	hexagonal
space group	R-3
Unit cell dimensions	a = 24.6217 Å α = 90 deg.
	b = 24.6217 Å β = 90 deg.
	c = 10.1014 Å γ = 120 deg.
Volume/Å^3	5303.3
Z	6
Absorption coefficient/mm^{-1}	0.509
F(000)	1956.0
Radiation	CuKα (λ = 1.54184)
2θ range for data collection/°	4.83 to 76.627 deg.
Index ranges	-31 ≤ h ≤ 31
	-31 ≤ k ≤ 31
	-12 ≤ l ≤ 12
Goodness-of-fit on F^2	0.974
Space group	R -3
Hall group	-R 3
Moiety formula	C42 H39 N3, 3(C0.67 H0.67) [+ solvent]
Sum formula	C44 H41 N3 [+ solvent]

Mr	611.80
Dx,g cm-3	1.149
Nref	2483
Data completeness	0.974
Theta(max)	76.627
R(reflections)	0.0966(1939)
wR2(reflections)	0.3048(2418)
S	1.002
Npar	143

5. X-ray crystal data of DPA[3]-a

X-ray crystallography of single crystal obtained by vapor diffusion of MeOH (2 mL) into solutions of **DPA[3]-a** (2 mg) in $CHCl_3$ (1 mL). CCDC number: 2015861.

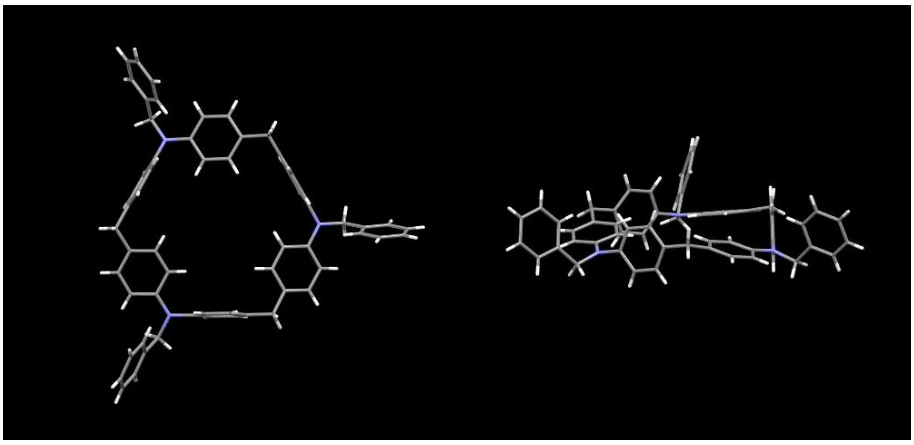

Supplementary Figure 53. X-ray crystal structure of **DPA[3]-a**.

Supplementary Table 10. Crystal data and structure refinement parameters for **DPA[3]-a**.

Identification code	exp_684
Empirical formula	$C_{20}H_{17}N$
Formula weight	271.35
Temperature/K	100.00(10)
Crystal system	trigonal
Space group	R-3
Unit cell dimensions	a = 23.58620 Å α = 90 deg.
	b = 23.58620 Å β = 90 deg.
	c = 15.99950 Å γ = 120 deg.
Volume/Å^3	7708.20(8)
Z	18
ρ_{calc}g/cm^3	1.052
μ/mm^{-1}	0.463
F(000)	2592.0
Crystal size/mm^3	0.48 × 0.42 × 0.36
Radiation	CuKα (λ = 1.54184)

2Θ range for data collection/°	7.018 to 149.046
Index ranges	$-29 \leq h \leq 29$
	$-29 \leq k \leq 29$
	$-19 \leq l \leq 19$
Reflections collected	65256
Independent reflections	3495 [R_{int} = 0.0507, R_{sigma} = 0.0153]
Data/restraints/parameters	3495/0/191
Goodness-of-fit on F^2	1.084
Final R indexes [I>=2σ (I)]	R_1 = 0.0448, wR_2 = 0.1137
Final R indexes [all data]	R_1 = 0.0463, wR_2 = 0.1147
Largest diff. peak/hole / e Å^{-3}	0.62/-0.26

6. X-ray crystal data of DPA[3]-b

X-ray crystallography of single crystal obtained by vapor diffusion of MeOH (2 mL) into solutions of **Bn-DPA[3]-b** (2 mg) in $CHCl_3$ (1 mL). CCDC number: 2011992.

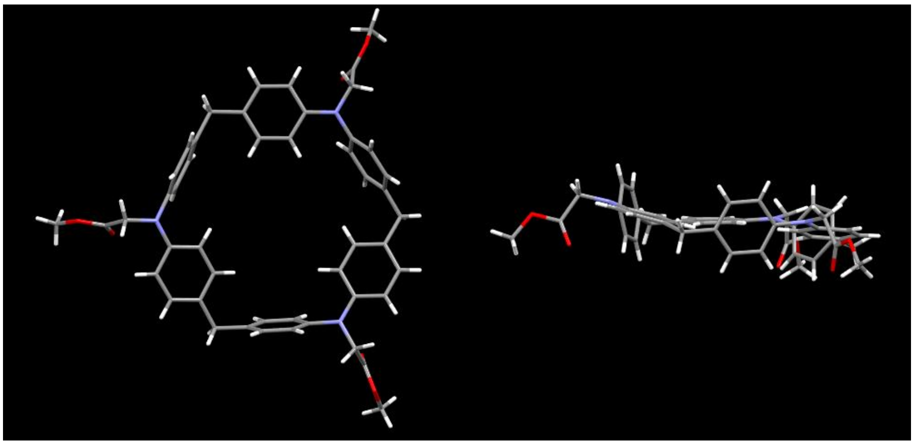

Supplementary Figure 54. X-ray crystal structure of **DPA[3]-b**.

Supplementary Table 11. Crystal data and structure refinement parameters for **DPA[3]-b**.

Identification code	exp_1175
Empirical formula	$C_{49}H_{46}Cl_3N_3O_6$
Formula weight	879.24
Temperature/K	100.00(10)
Crystal system	monoclinic
Space group	$P2_1/c$
Unit cell dimensions	a = 18.1858 Å α = 90 deg.
	b = 26.5500 Å β = 90 deg.
	c = 9.9665 Å γ = 120 deg.
Volume/Å^3	4805.48(12)
Z	4
ρ_{calc}g/cm^3	1.215
μ.21^{-1}	2.122
F(000)	1840.0

Crystal size/mm^3	0.39 al size/mmlad
Radiation	CuKαuKiationize/mm
2uKiationize/mmladetructure re	5.896 to 134.15
Index ranges	$-21 \leq h \leq 21$
	$-31 \leq k \leq 31$
	$-11 \leq l \leq 11$
Reflections collected	120717
Independent reflections	8541 [R_{int} = 0.0775, R_{sigma} = 0.0320]
Data/restraints/parameters	8541/0/553
Goodness-of-fit on F^2	1.032
Final R indexes [I>=2eters,	R_1 = 0.0829, wR_2 = 0.2163
Final R indexes [all data]	R_1 = 0.0925, wR_2 = 0.2238
Largest diff. peak/hole / e Å^{-3}	1.13/-1.01

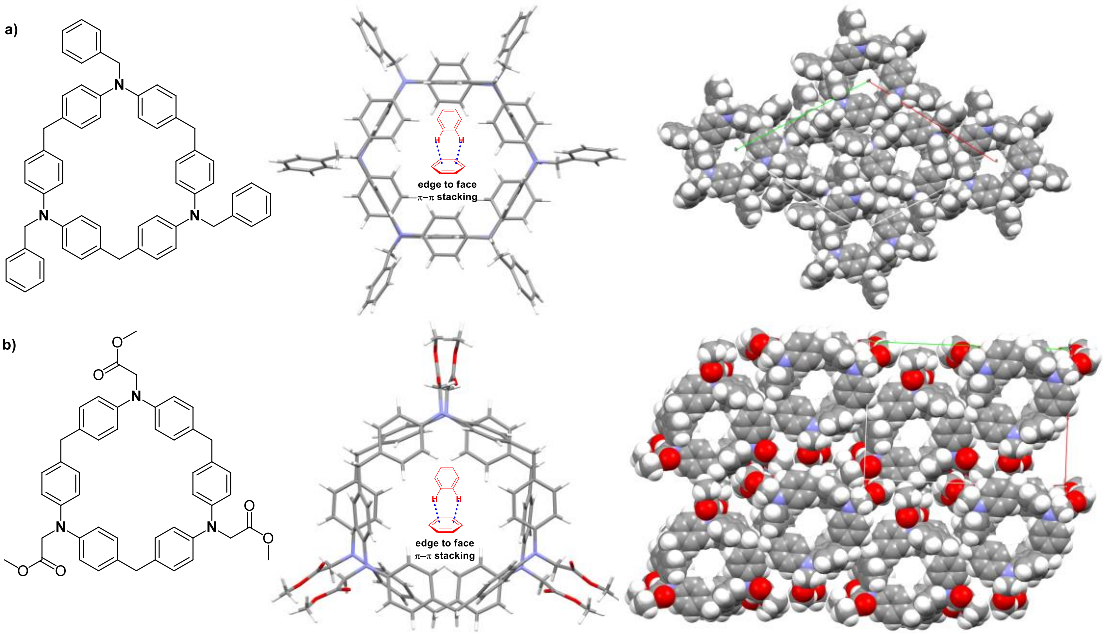

Supplementary Figure 55. The edge to face π-π stacking motif and columnar self-assembling behaviors of (a) **DPA[3]-a** and (b) **DPA[3]-b**.

Section H. Characterization data and spectra for DPA[n]s

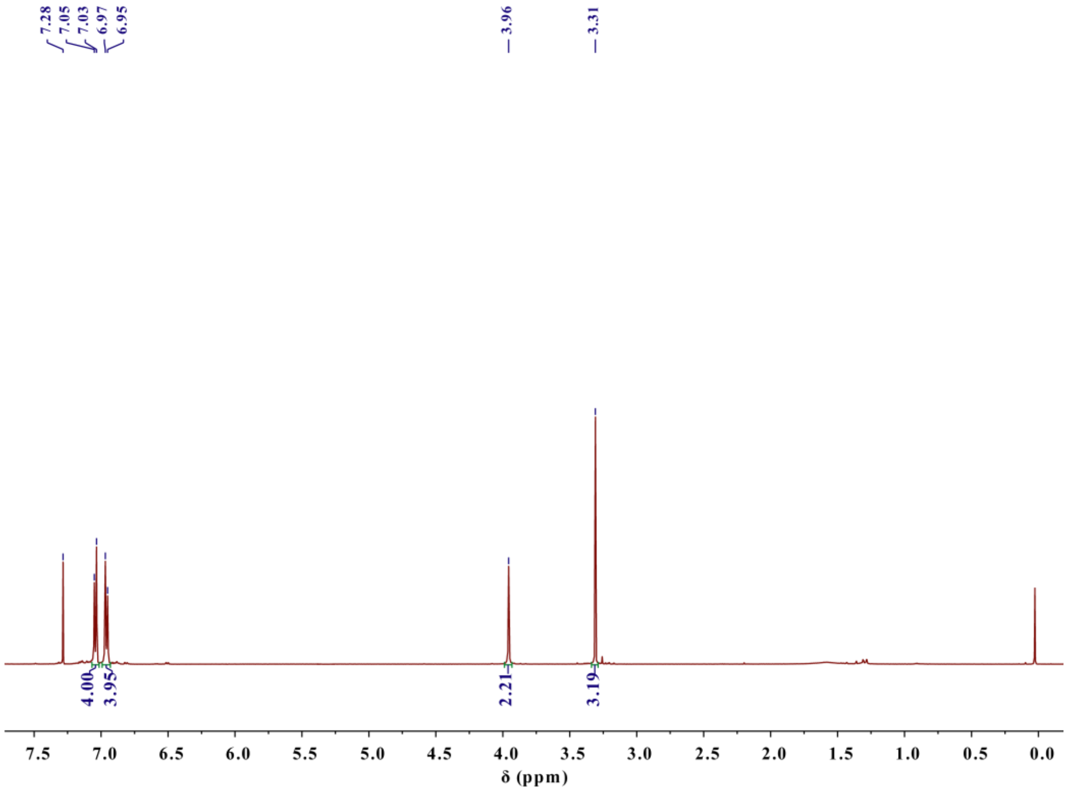

Supplementary Figure 56. ^{1}H NMR spectrum (400 MHz, 298 K) of **DPA[3]** in $CDCl_3$.

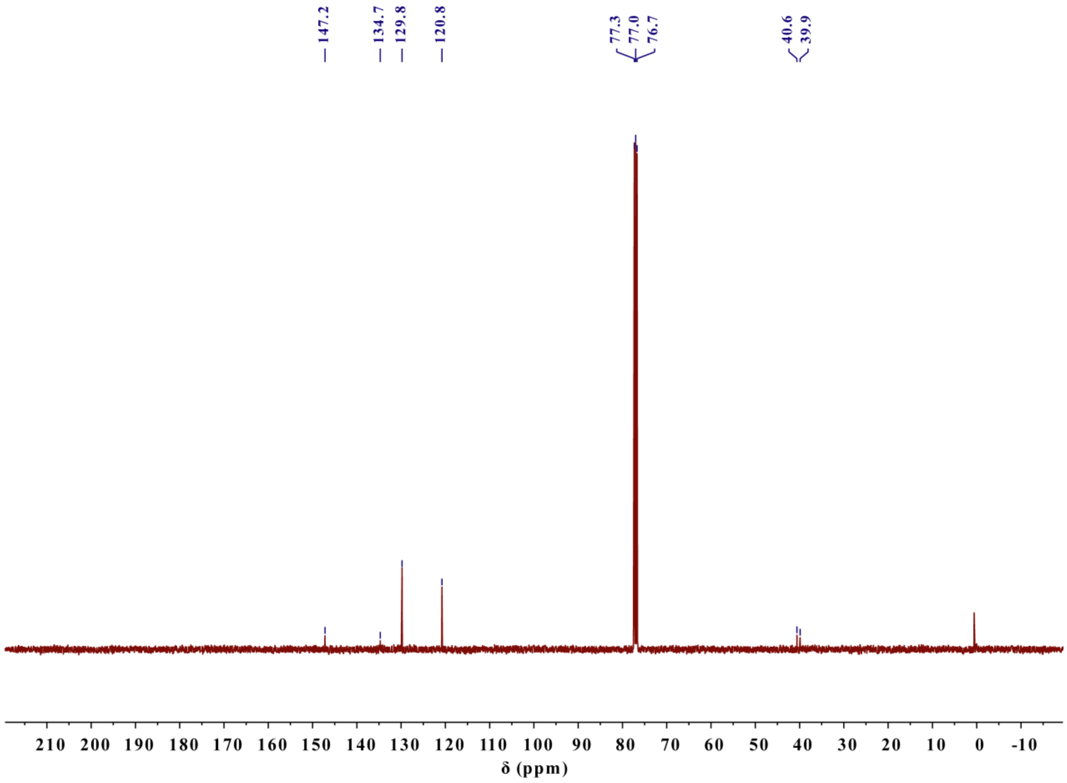

Supplementary Figure 57. ^{13}C NMR spectrum (100 MHz, 298 K) of **DPA[3]** in $CDCl_3$.

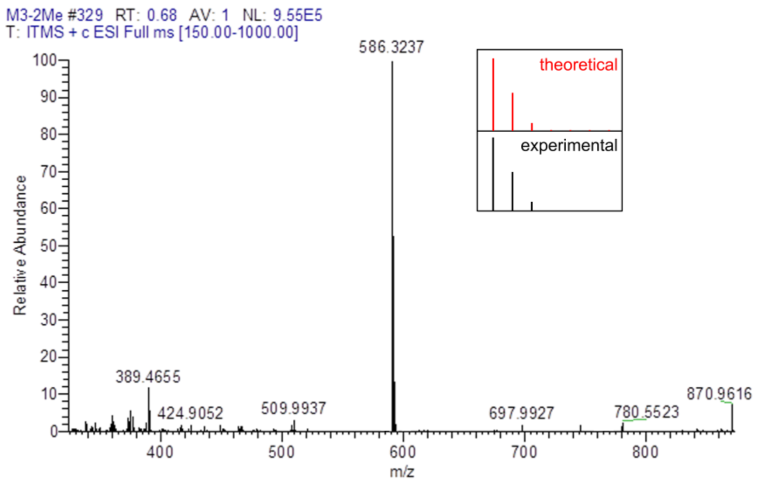

Supplementary Figure 58. HR-ESI-TOF MS spectrum of **DPA[3]** and the isotope distribution.

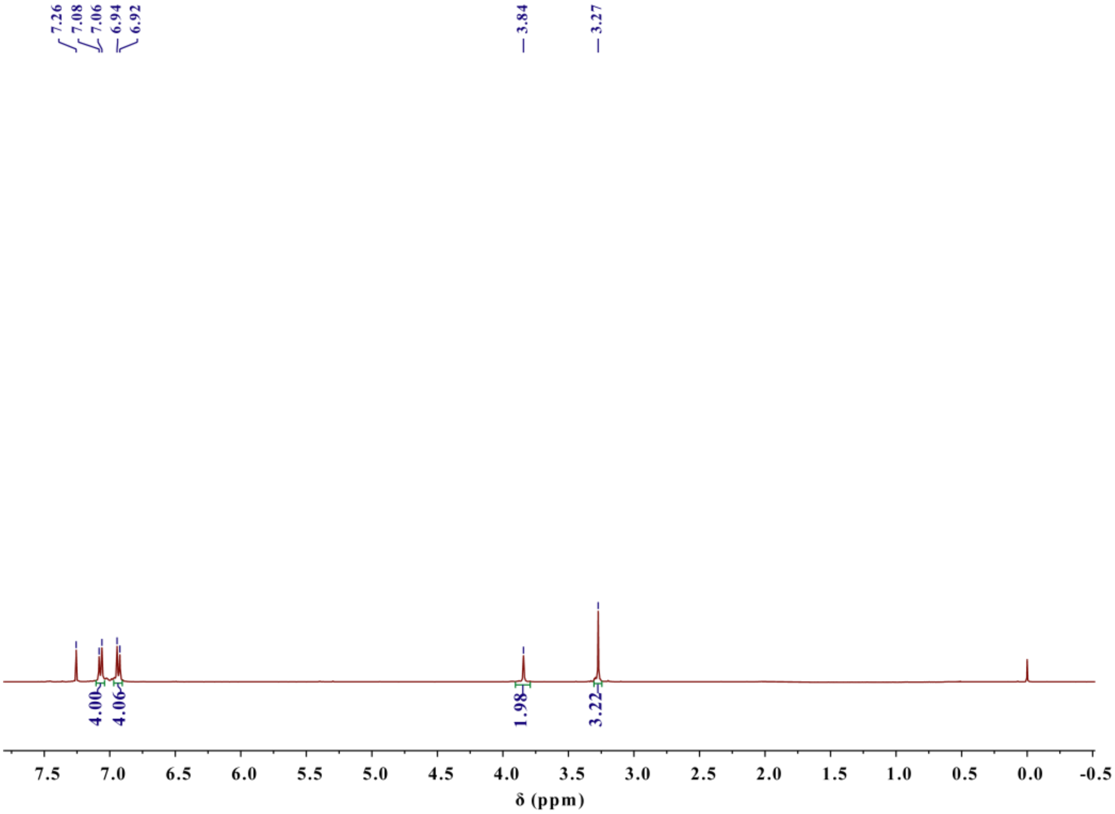

Supplementary Figure 59. ^{1}H NMR spectrum (400 MHz, 298 K) of **DPA[4]** in $CDCl_3$.

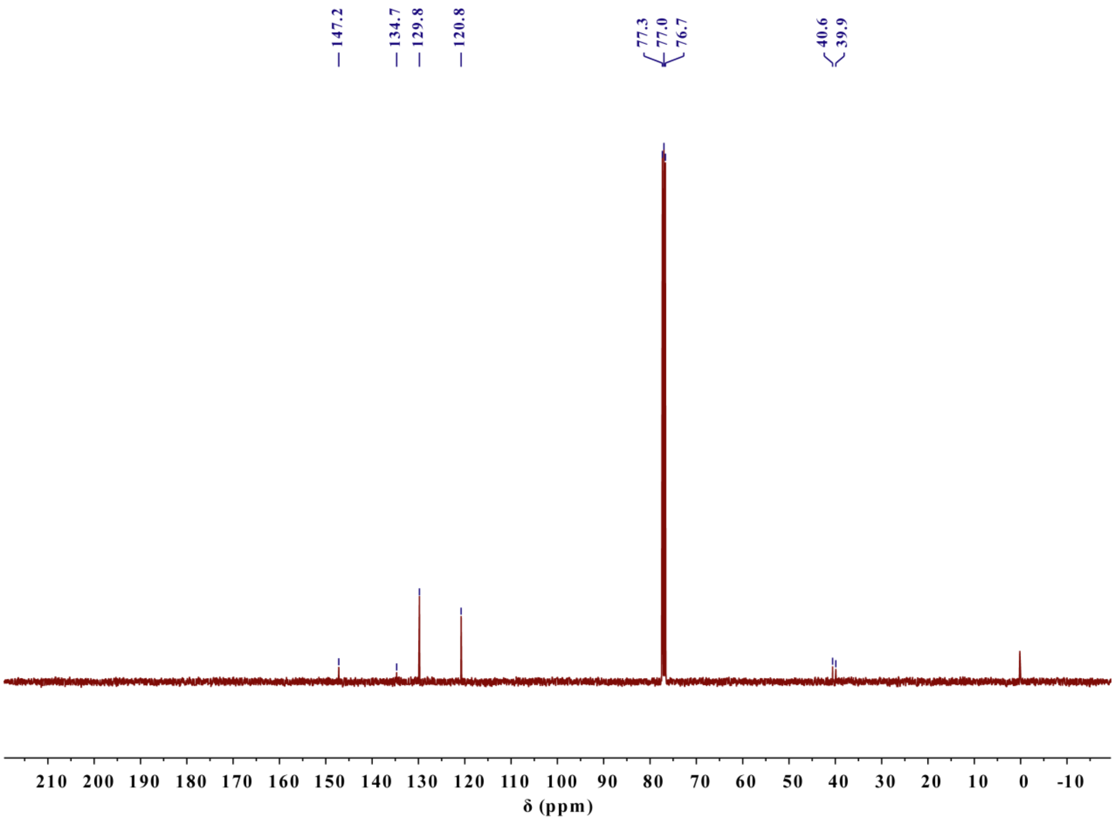

Supplementary Figure 60. ^{13}C NMR spectrum (100 MHz, 298 K) of **DPA[4]** in $CDCl_3$.

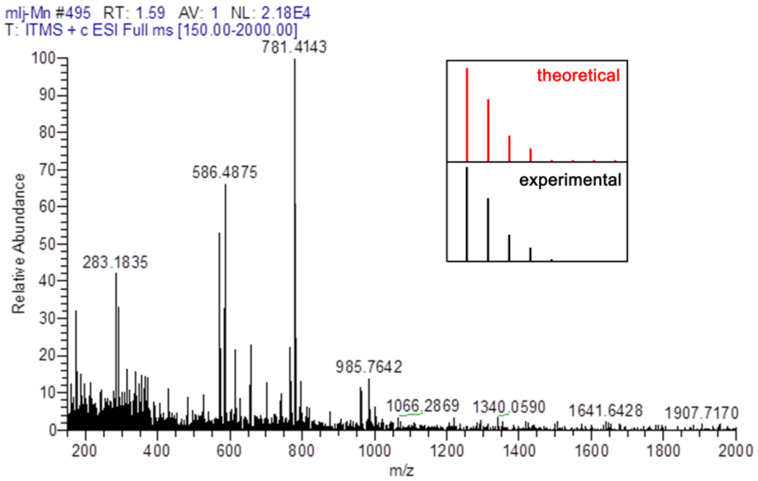

Supplementary Figure 61. HR-ESI-TOF MS spectrum of **DPA[4]** and the isotope distribution.

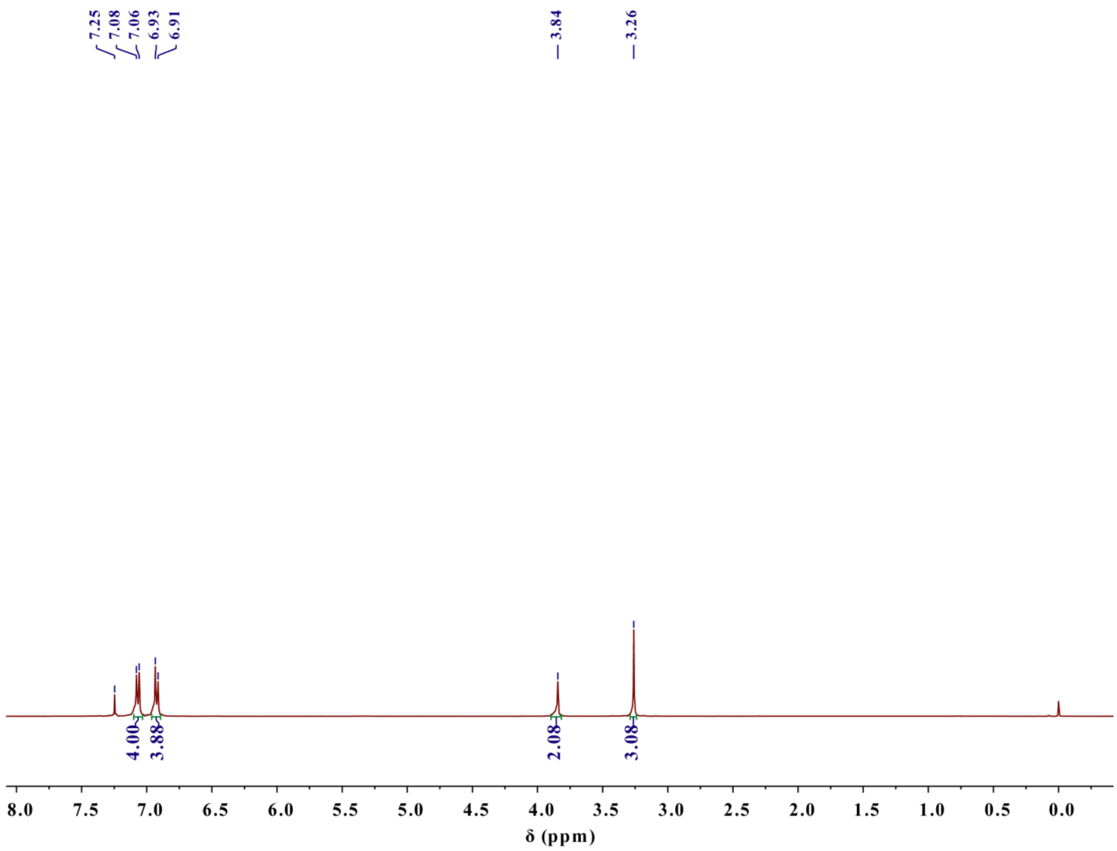

Supplementary Figure 62. ^{1}H NMR spectrum (400 MHz, 298 K) of **DPA[5]** in $CDCl_3$.

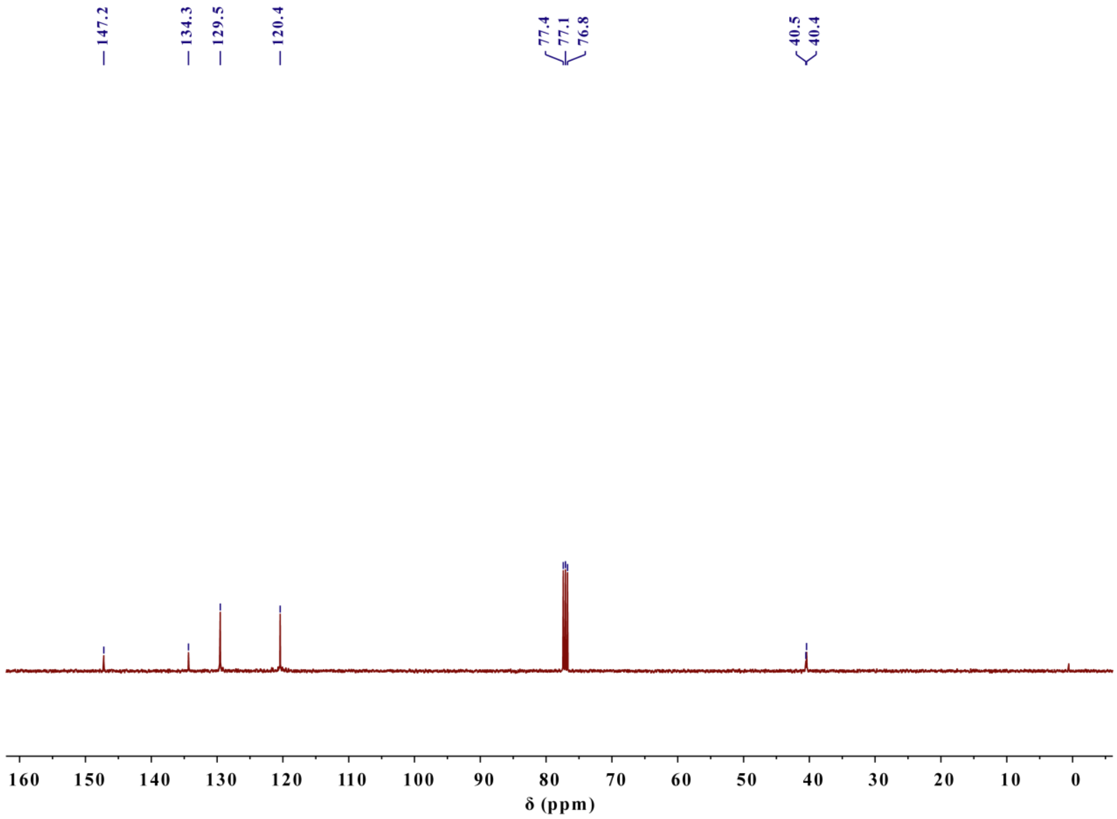

Supplementary Figure 63. ^{13}C NMR spectrum (100 MHz, 298 K) of **DPA[5]** in $CDCl_3$.

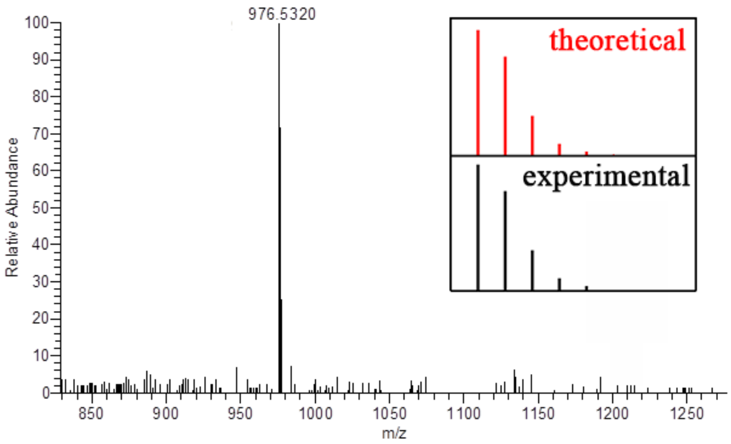

Supplementary Figure 64. HR-ESI-TOF MS spectrum of **DPA[5]** and the isotope distribution.

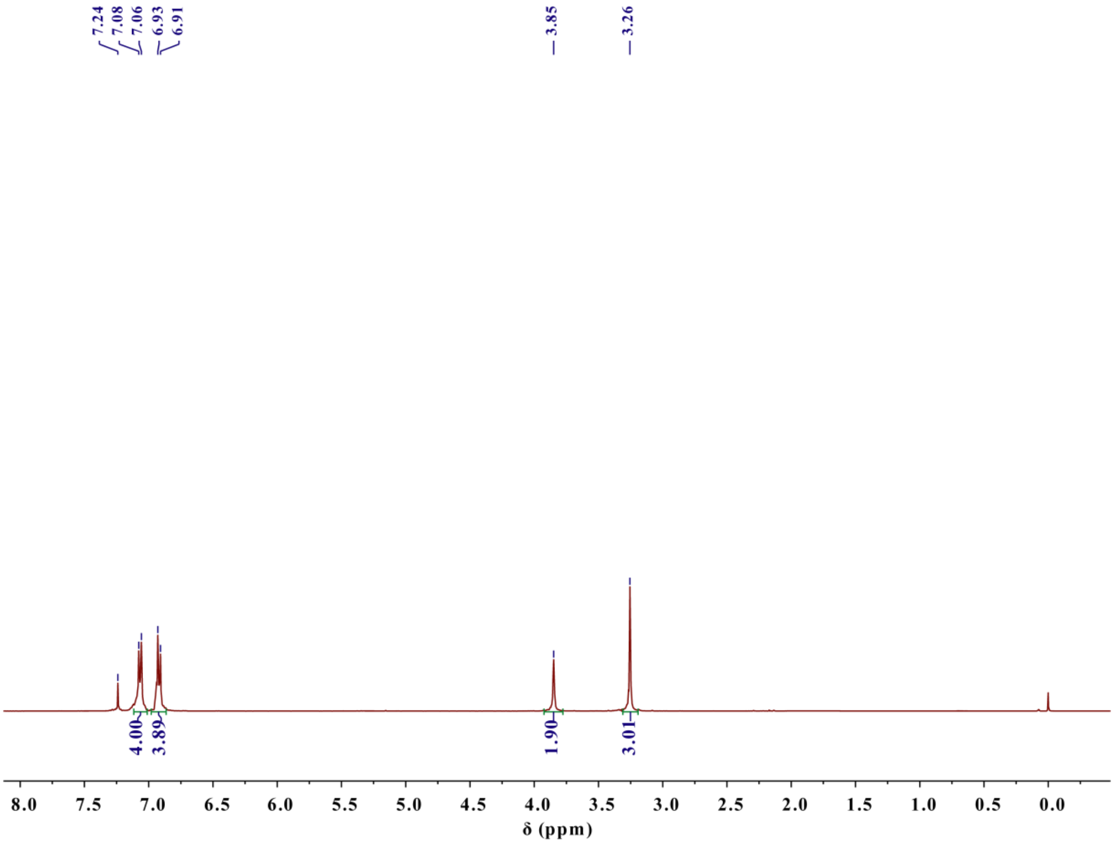

Supplementary Figure 65. ^{1}H NMR spectrum (400 MHz, 298 K) of **DPA[6]** in $CDCl_3$.

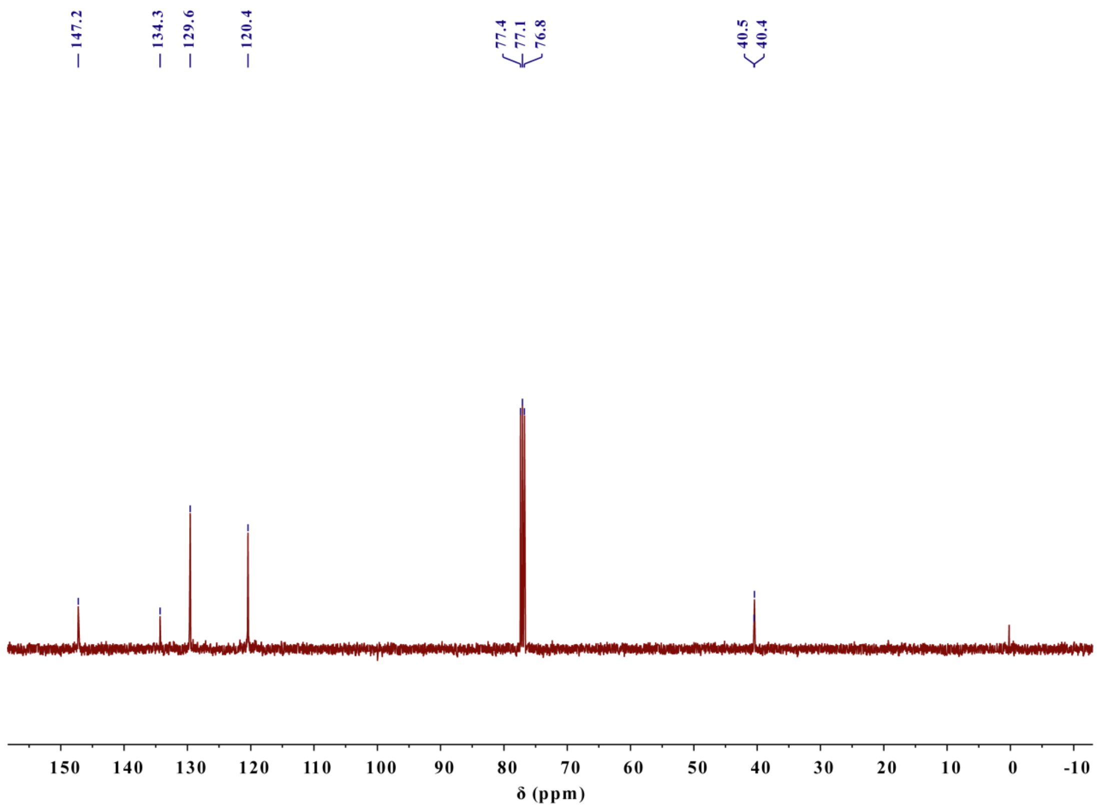

Supplementary Figure 66. ^{13}C NMR spectrum (100 MHz, 298 K) of **DPA[6]** in $CDCl_3$.

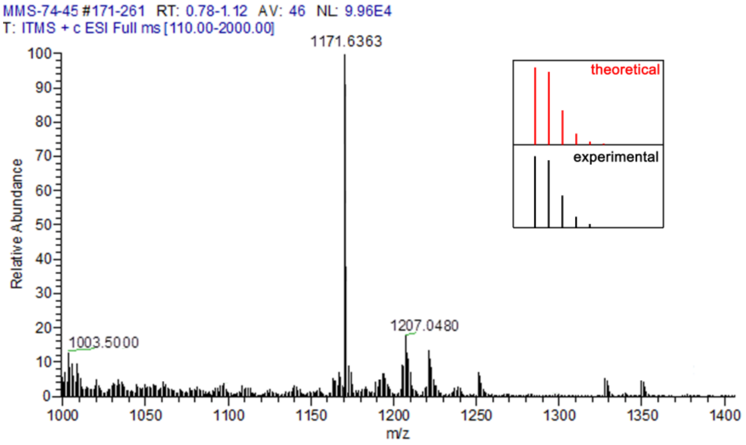

Supplementary Figure 67. HR-ESI-TOF MS spectrum of **DPA[6]** and the isotope distribution.

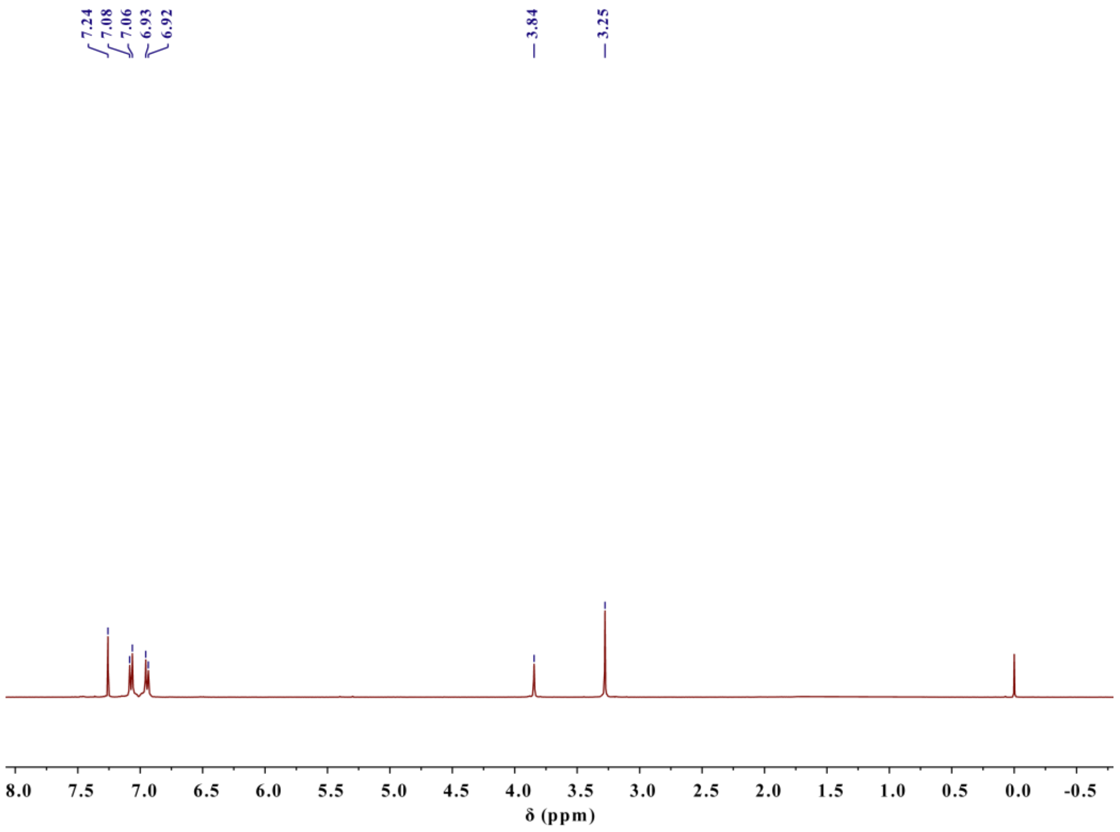

Supplementary Figure 68. ^{1}H NMR spectrum (400 MHz, 298 K) of **DPA[7]** in $CDCl_3$.

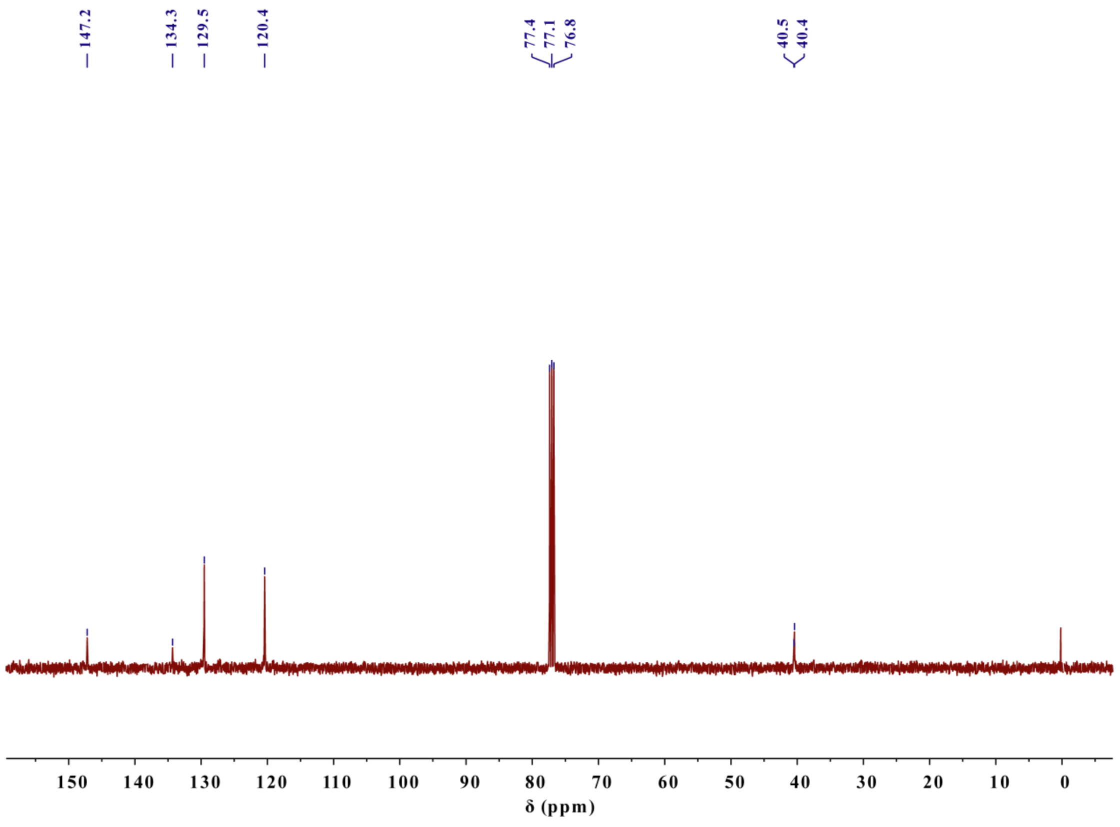

Supplementary Figure 69. ^{13}C NMR spectrum (100 MHz, 298 K) of **DPA[7]** in $CDCl_3$.

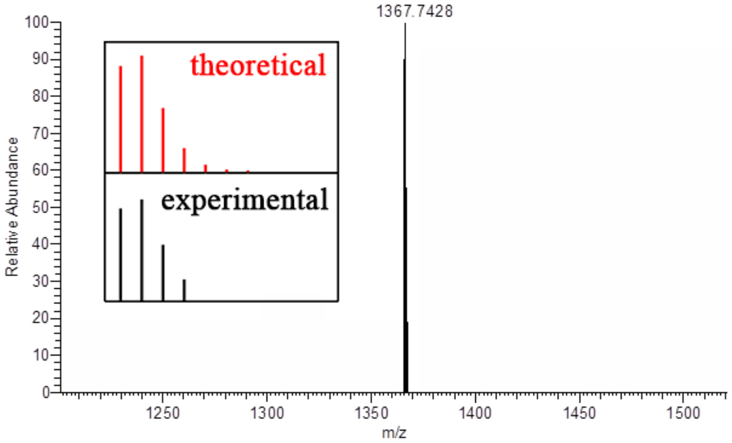

Supplementary Figure 70. HR-ESI-TOF MS spectrum of **DPA[7]** and the isotope distribution.

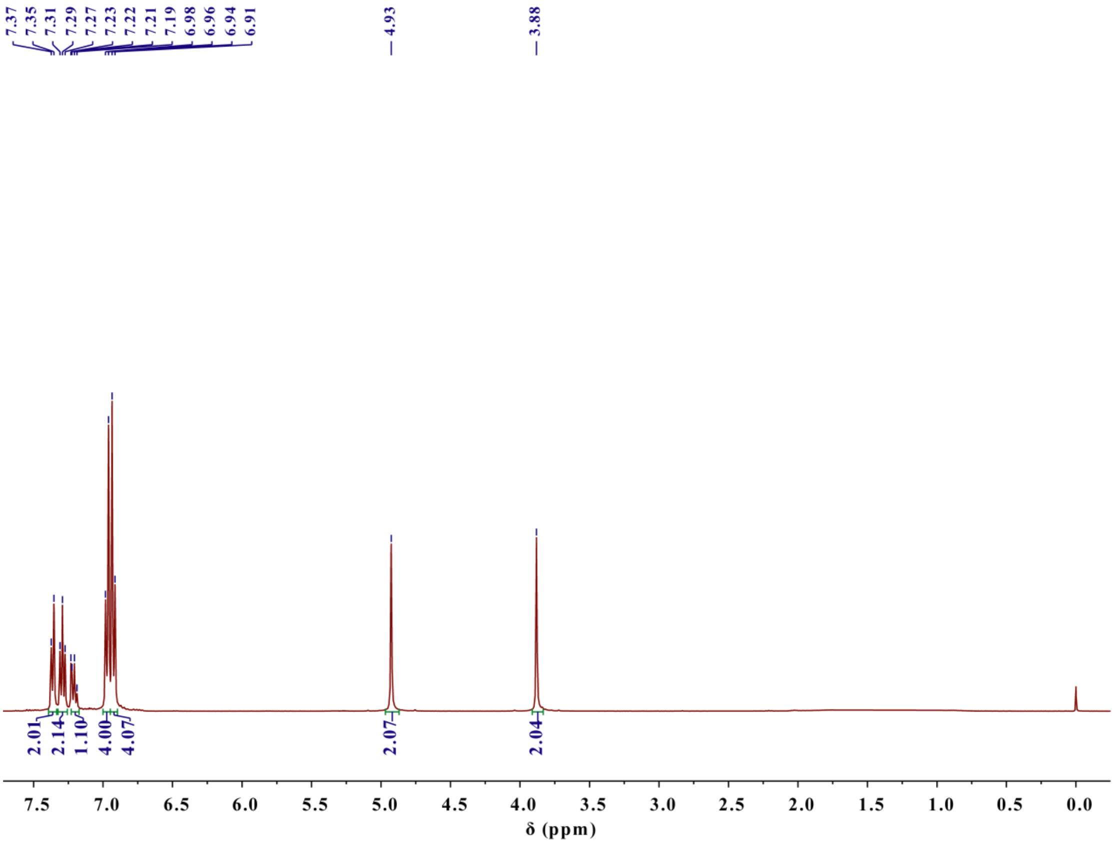

Supplementary Figure 71. ^{1}H NMR spectrum (400 MHz, 298 K) of **DPA[3]-a** in $CDCl_3$.

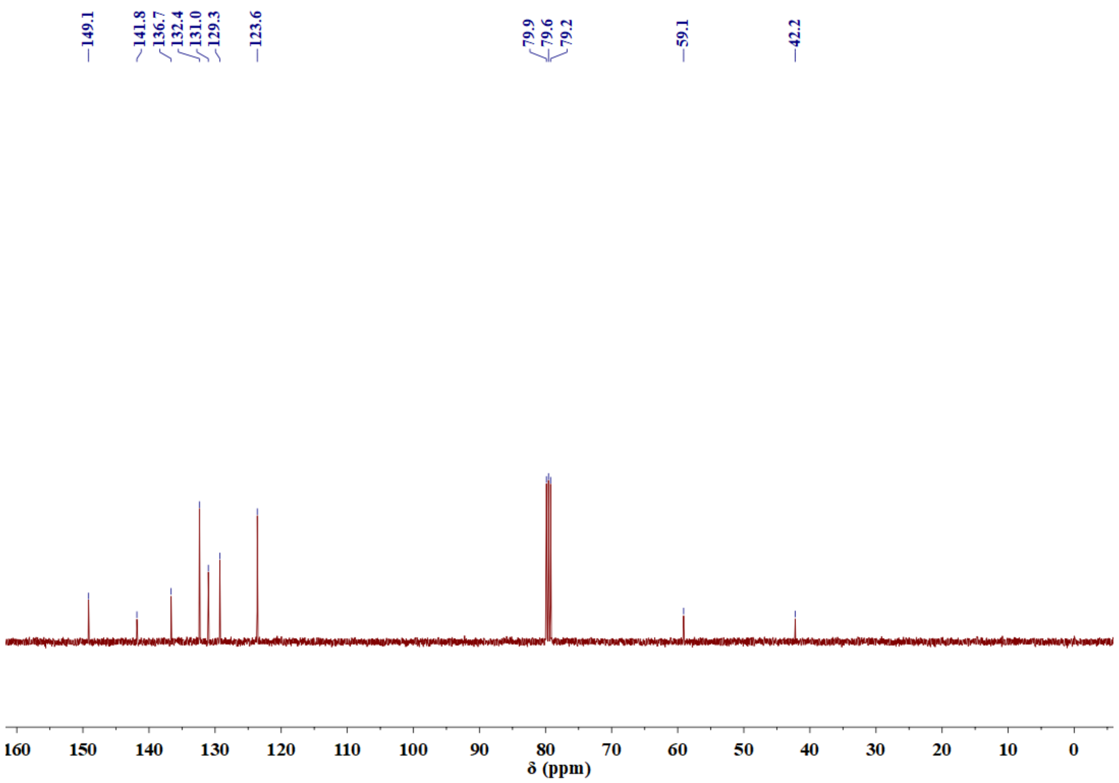

Supplementary Figure 72. ^{13}C NMR spectrum (100 MHz, 298 K) of **DPA[3]-a** in $CDCl_3$.

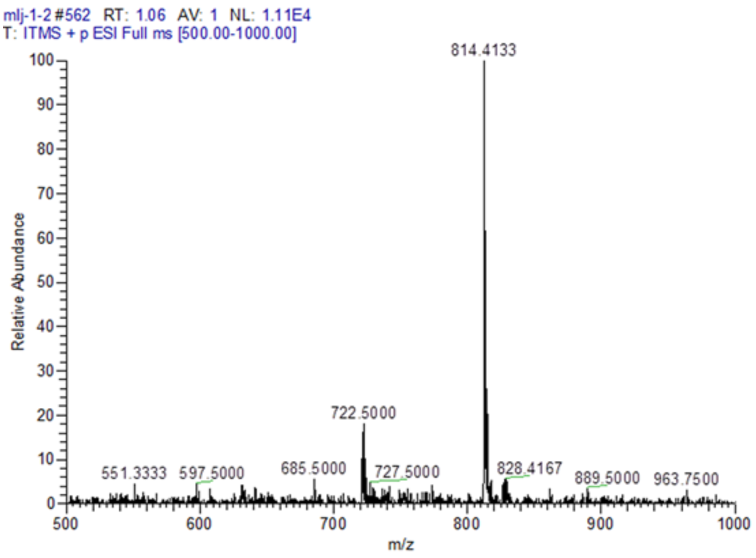

Supplementary Figure 73. HR-ESI-TOF MS spectrum of **DPA[3]-a**.

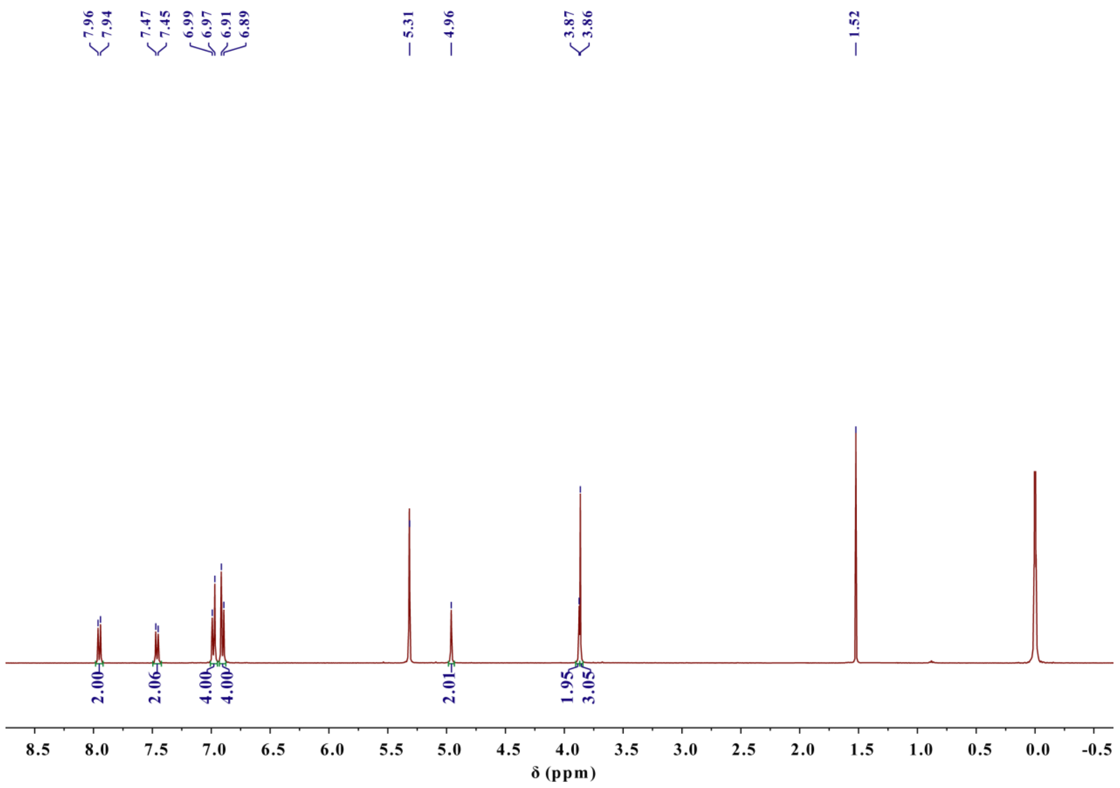

Supplementary Figure 74. ^{1}H NMR spectrum (400 MHz, 298 K) of **DPA[3]-b** in CD_2Cl_2.

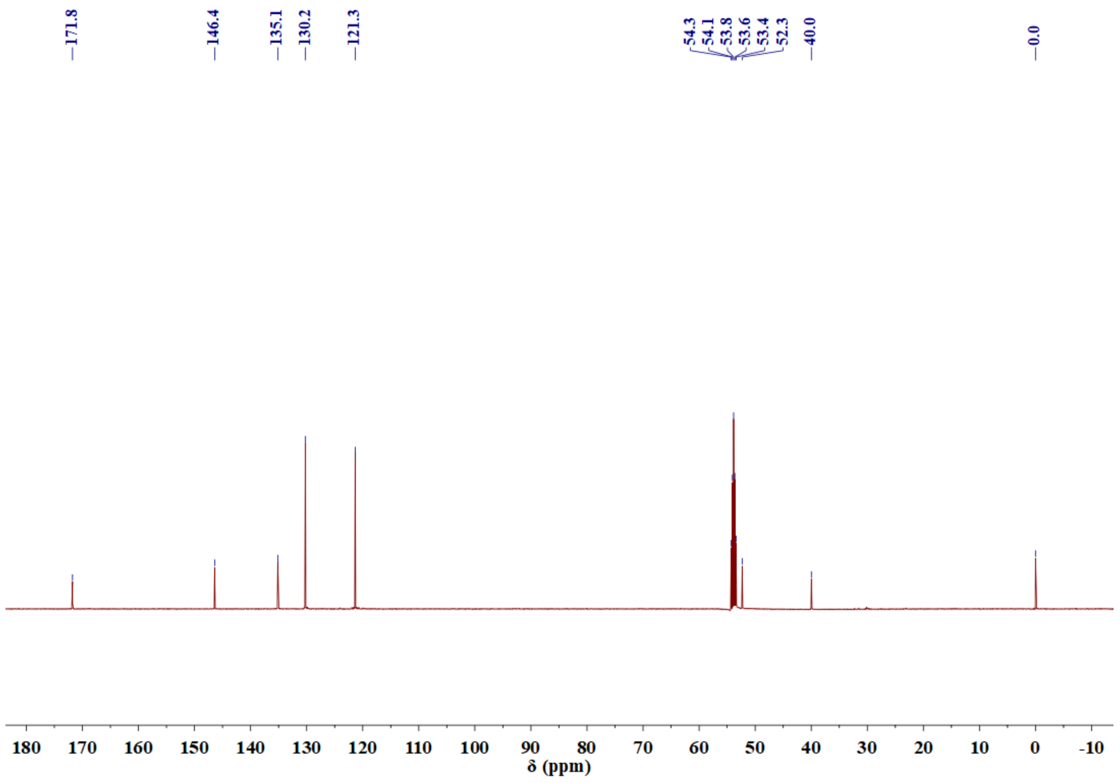

Supplementary Figure 75. ^{13}C NMR spectrum (100 MHz, 298 K) of **DPA[3]-b** in CD_2Cl_2.

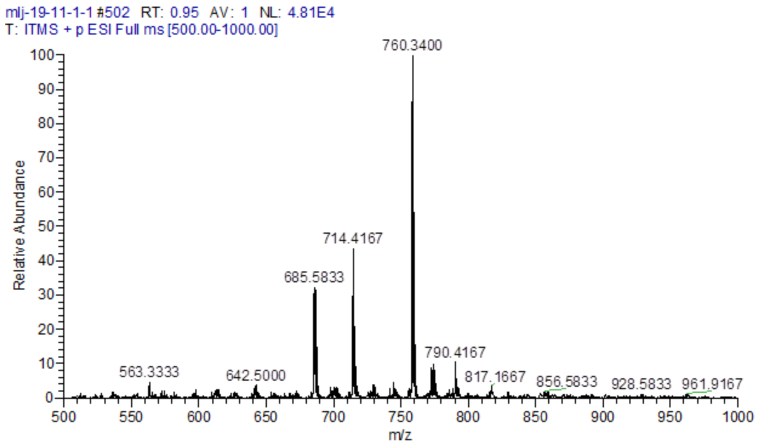

Supplementary Figure 76. HR-ESI-TOF MS spectrum of **DPA[3]-b**.

Section I. Reference

1. Peng, C. & Schlegel, H. B. Combining synchronous transit and quasi-newton methods to find transition states. *Israel J. Chem.* **33**, 449-454 (1993).

2. Peng, C., Ayala, P. Y., Schlegel, H. B. & Frisch, M. J. Using redundant internal coordinates to optimize equilibrium geometries and transition states. *J. Comp. Chem.* **17**, 49-56 (1996).

3. 6. Gaussian 16, Revision A.03, M. J. Frisch, G. W. Trucks, H. B. Schlegel, G. E. Scuseria, M. A. Robb, J. R. Cheeseman, G. Scalmani, V. Barone, G. A. Petersson, H. Nakatsuji, X. Li, M. Caricato, A. V. Marenich, J. Bloino, B. G. Janesko, R. Gomperts, B. Mennucci, H. P. Hratchian, J. V. Ortiz, A. F. Izmaylov, J. L. Sonnenberg, D. Williams-Young, F. Ding, F. Lipparini, F. Egidi, J. Goings, B. Peng, A. Petrone, T. Henderson, D. Ranasinghe, V. G. Zakrzewski, J. Gao, N. Rega, G. Zheng, W. Liang, M. Hada, M. Ehara, K. Toyota, R. Fukuda, J. Hasegawa, M. Ishida, T. Nakajima, Y. Honda, O. Kitao, H. Nakai, T. Vreven, K. Throssell, J. A. Montgomery, Jr., J. E. Peralta, F. Ogliaro, M. J. Bearpark, J. J. Heyd, E. N. Brothers, K. N. Kudin, V. N. Staroverov, T. A. Keith, R. Kobayashi, J. Normand, K. Raghavachari, A. P. Rendell, J. C. Burant, S. S. Iyengar, J. Tomasi, M. Cossi, J. M. Millam, M. Klene, C. Adamo, R. Cammi, J. W. Ochterski, R. L. Martin, K. Morokuma, O. Farkas, J. B. Foresman, and D. J. Fox, Gaussian, Inc., Wallingford CT, 2016.

4. Grimme, S. Semiempirical GGA-type density functional constructed with a long-range dispersion correction. *J. Comp. Chem.* **27**, 1787-1799 (2006).
